# A Positive Feedback Loop Involving Gcm1 and Fzd5 Directs Chorionic Branching Morphogenesis in the Placenta

**DOI:** 10.1371/journal.pbio.1001536

**Published:** 2013-04-16

**Authors:** Jinhua Lu, Shuang Zhang, Haruo Nakano, David G. Simmons, Shumin Wang, Shuangbo Kong, Qiang Wang, Lianju Shen, Zhaowei Tu, Weixiang Wang, Bingyan Wang, Hongmei Wang, Yanling Wang, Johan H. van Es, Hans Clevers, Gustavo Leone, James C. Cross, Haibin Wang

**Affiliations:** 1 State Key Laboratory of Reproductive Biology, Institute of Zoology, Chinese Academy of Sciences, Beijing, People's Republic of China; 2 Graduate School of the Chinese Academy of Sciences, Beijing, People's Republic of China; 3 Department of Comparative Biology and Experimental Medicine, Faculty of Veterinary Medicine, University of Calgary, Calgary, Alberta, Canada; 4 Hubrecht Institute, Netherlands Institute for Developmental Biology, Utrecht, The Netherlands; 5 Departments of Molecular Virology, Immunology, and Medical Genetics, Human Cancer Genetics Program, College of Medicine, Comprehensive Cancer Center, The Ohio State University, Columbus, Ohio, United States of America; The Hospital for Sick Children, Canada

## Abstract

Chorioallantoic branching morphogenesis is a key milestone during placental development, creating the large surface area for nutrient and gas exchange, and is therefore critical for the success of term pregnancy. Several Wnt pathway molecules have been shown to regulate placental development. However, it remains largely unknown how Wnt-Frizzled (Fzd) signaling spatiotemporally interacts with other essential regulators, ensuring chorionic branching morphogenesis and angiogenesis during placental development. Employing global and trophoblast-specific *Fzd5*-null and *Gcm1*-deficient mouse models, combining trophoblast stem cell lines and tetraploid aggregation assay, we demonstrate here that an amplifying signaling loop between Gcm1 and Fzd5 is essential for normal initiation of branching in the chorionic plate. While *Gcm1* upregulates *Fzd5* specifically at sites where branching initiates in the basal chorion, this elevated *Fzd5* expression via nuclear β-catenin signaling in turn maintains expression of *Gcm1*. Moreover, we show that Fzd5-mediated signaling induces the disassociation of cell junctions for branching initiation via downregulating ZO-1, claudin 4, and claudin 7 expressions in trophoblast cells at the base of the chorion. In addition, Fzd5-mediated signaling is also important for upregulation of *Vegf* expression in chorion trophoblast cells. Finally, we demonstrate that Fzd5-Gcm1 signaling cascade is operative during human trophoblast differentiation. These data indicate that Gcm1 and Fzd5 function in an evolutionary conserved positive feedback loop that regulates trophoblast differentiation and sites of chorionic branching morphogenesis.

## Abstract 2

Placenta formation during pregnancy requires chorioallantoic branching morphogenesis that involves establishing an amplifying feedback loop between Frizzled5 and Gcm1 to regulate branching initiation and trophoblast differentiation.

## Author Summary

Abnormal placental development during pregnancy is associated with conditions such as preeclampsia, intrauterine growth restriction, and even fetal death in humans. Here we focus on the earliest steps of placenta formation, which involves the development of the labyrinthine layer, a specialized epithelium that sits between the maternal blood and fetal blood vessels and facilitates the exchange of nutrients, gases, and wastes between the mother and fetus. Pivotal to the development of a functional labyrinth layer are the processes of folding and branching of a flat sheet of trophoblast cells (originally the outer layer of the blastocyst), and of trophoblast cell differentiation. Here, we show in mice that Frizzled5, a receptor component of the Wnt signaling pathway, and Gcm1, an important transcription factor for labyrinth development, form a positive feedback loop that directs normal placental development. We find that *Gcm1* up-regulates *Fzd5* specifically at branching sites and that elevated *Fzd5* expression in turn maintains expression of *Gcm1*. Moreover, Fzd5-mediated signaling is required for the disassociation of cell junctions and for the up-regulation of *Vegf* expression in trophoblast cells. Finally, with implications for human disease, we demonstrate that the FZD5-GCM1 signaling cascade operates in primary cultures of human trophoblasts undergoing differentiation.

## Introduction

The placenta is a temporary organ first formed during pregnancy that is essential for the survival and growth of the fetus in eutherian mammals. Abnormal placental development is often associated with intrauterine growth restriction, preeclampsia, and even fetal death in humans [1]–[3]. The development of placenta starts at embryonic day 4.5 (E4.5) in mice, when the formation of different trophoblast cell types is underway. By around E10.5, a placenta with complete structure has formed. The mature placenta is composed of three major layers: the outermost layer is comprised of trophoblast giant cells and is adjacent to maternal decidua; spongiotrophoblast cells form a layer between the labyrinth and outer giant cells, and the innermost layer is the labyrinth layer, a layer important for the exchange of nutrients, gases, and wastes between the mother and fetus. Development of the labyrinth is divided into three stages: chorioallantoic attachment at E8.5, initiation of branching in trophoblast cells at the base of the chorionic plate, and branching morphogenesis and vascularization in the chorionic plate. Disturbance to any one of these stages would lead to an impaired labyrinth development, resulting in failure of pregnancy. The *Glial cells missing–1* (*Gcm1*) gene lies at a key step in labyrinth development, since its expression in clusters of trophoblast cells at the base of the chorion define the initiation of branchpoints [4]. While Gcm1 expression appears autonomously before chorioallantoic attachment, the maintenance of its expression during subsequent branching is dependent on contact with the allantois [5]. The signals that establish the initial Gcm1 pattern and which maintain it have not yet been defined.

Gene targeting experiments have shown that development of labyrinth is regulated by numerous signaling molecules [1]–[3] including the Wnt signaling pathway [6]. For example, mice with null mutation of *Wnt7b*, expressed in the chorion, die at mid-gestation stages due to a defect of chorioallantoic attachment [7]. Mutation of *R-spondin3*, a molecule that promotes the Wnt-β-catenin signaling pathway, leads to failure of branchpoint initiation in the chorionic plate [8]. Similarly, deletion of *Bcl9l*, a vertebrate ortholog of *Drosophila legless* and an essential intracellular member of Wnt pathway, also results in defective branchpoint initiation and impaired differentiation of trophoblast cells in the chorion into syncytiotrophoblast layer II (SynT-II) cells [9]. Moreover, targeted disruption of *Wnt2* causes an impaired development of the labyrinth at a slightly later stage of gestation but still leading to perinatal embryo demise [10]. Defective labyrinth development has also been reported in *Frizzled5* (*Fzd5*) mutant mice [11] though the details of the phenotype have not yet been reported. Therefore, it remains largely unknown how different Wnt ligands signal via Fzd5 receptors to regulate chorionic branching morphogenesis and/or vascularization of the labyrinth. Moreover, it is unknown how Wnt-Fzd5 signaling interacts with other essential regulators during the development of labyrinth.

In the present study, we have employed a variety of in vivo and in vitro models to address how Fzd5 regulates chorioallantoic development during placentation. We provide direct genetic evidence that an amplifying signaling hierarchy between Gcm1 and Fzd5 directs branching morphogenesis and trophoblast differentiation during placental development in both mice and humans.

## Results

### Fzd5 Is Expressed in a Spatiotemporally Restricted Manner in the Developing Labyrinth Layer of the Placenta

**Figure 1:**
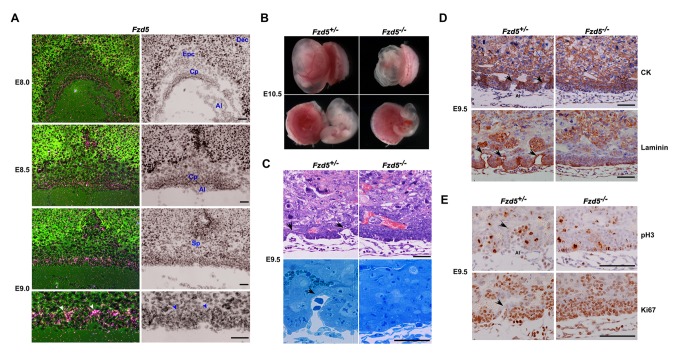
*Fzd5* is spatiotemporally expressed in the developing placenta and its deficiency derails normal initiation of branching in the chorion. (A) Expression of *Fzd5* in trophoblast cells of the chorion during early placentation from E8.0–9.0 by in situ hybridization with high expression at the tips of branchpoints in the E9.0 chorionic plate (white arrowheads). In addition, low levels of *Fzd5* mRNA were also detected in the floating allantois at E8.0, from which the fetal vessels in the labyrinth are derived. (B) Whole mount views of E10.5 control (+/−) and *Fzd5*-null (−/−) placentas and yolk sacs. *Fzd5*-null embryos showed growth retardation and pale yolk sacs. (C) Hematoxylin-eosin (HE) staining and Toluidine blue staining (semithin section) of E9.5 control (+/−) and *Fzd5*-null (−/−) chorionic plates. Note that the chorion has begun to branch and form primary villi with associated blood vessels from the allantois in controls (+/−) (black arrows), but this did not happen in *Fzd5* nulls (−/−). (D) Immunostaining of cytokeratin (CK) and laminin in E9.5 control (+/−) and *Fzd5*-null (−/−) placentas. Cytokeratin marks placental trophoblast cells and laminin outlines the fetal vascular endothelial cells. (E) The proliferation state of E9.5 control (+/−) and *Fzd5*-null (−/−) chorion plate revealed by Ki67 and pH3 immunostaining. Ki67 and pH3 positive cells are in the state of proliferation. Note that trophoblast cells lining the branchpoint sites in the chorion plate ceased proliferation in control (+/−) chorion (black arrows), but not in Fzd5-null (−/−) chorion. Al, allantois; Cp, Chorionic plate; Dec, decidua; Epc, ectoplacental core; Sp, spongiotrophoblast layer. Scale bars: 200 µm.

To explore the pathophysiological significance of Fzd5-driven signaling during placental branching morphogenesis, we first performed in situ hybridization to examine the spatiotemporal expression profile of Fzd5 receptors in the developing placenta. *Fzd5* mRNA expression was mainly detected in trophoblast cells of the chorion at E8.0, and was strikingly high at the branching points in the chorion at E9.0 (Figure 1A and Figure S1). Low levels of *Fzd5* mRNA were also detected in the floating allantois at E8.0, from which the fetal vessels in the labyrinth are derived; its expression declined to undetectable levels in the allantois upon attachment with the chorion at E8.5 (Figure 1A and Figure S1). *Fzd5* was also expressed in the yolk sac at later developmental stages (Figure S1), consistent with a previous report ascribing its necessity during yolk sac angiogenesis [11]. This spatiotemporal expression profile of *Fzd5* suggests that Fzd5-coupled signaling may play a role during early placental labyrinth development.

### Fzd5 Deficiency Derails the Normal Initiation of Branching Morphogenesis

To unveil the physiological significance of Fzd5 during chorionic villus development, we employed global *Fzd5*-null mutant mouse models achieved by crossing *Fzd5^loxp/loxp^* mice [12] with *Zp3-Cre* ^+/−^ mice. The yolk sacs of *Fzd5*-null mutant placentas at E10.5 were pale and devoid of blood vessels, with severely retarded fetal growth (Figure 1B). These defects are consistent with previous observations showing that *Fzd5* is essential for yolk sac angiogenesis [11]. In addition to changes in the yolk sac, the labyrinth layer of the placenta was also significantly underdeveloped. Attachment of the chorion and allantois occurred normally in *Fzd5* mutants with normal expression of vascular cell adhesion molecule–1 (VCAM-1) and α4 integrin at E8.5 (Figure S2), which are required for chorioallantoic attachment [13]–[15]. However, the initiation and progression of branching morphogenesis in the chorion failed to occur at E9.5 in *Fzd5* mutants (Figure 1C). Immunostaining analysis of cytokeratin, which marks the placental trophoblast cells, and laminin, which stains the blood vessel endothelial cells, clearly revealed that the chorion remained flat and the primary villous branches did not initiate (Figure 1D).

The defective chorioallantoic branching was associated with altered trophoblast proliferation and differentiation. In control (*Fzd5* ^+/−^) placentas, trophoblast cells lining the branchpoint site in the chorionic plate ceased proliferation and mitotic division, showing no staining for either Ki67 or phospho-histone H3 (pH3), consistent with previous findings [16]. By contrast, most trophoblast cells within the *Fzd5* ^−/−^ chorion still underwent proliferation showing positive staining for Ki67 and pH3 (Figure 1E), reinforcing the notion that *Fzd5* deficiency derails the normal initiation of branching morphogenesis.

### Trophoblast-Expressed Fzd5 Is Essential for Chorioallantoic Branching

**Figure 2:**
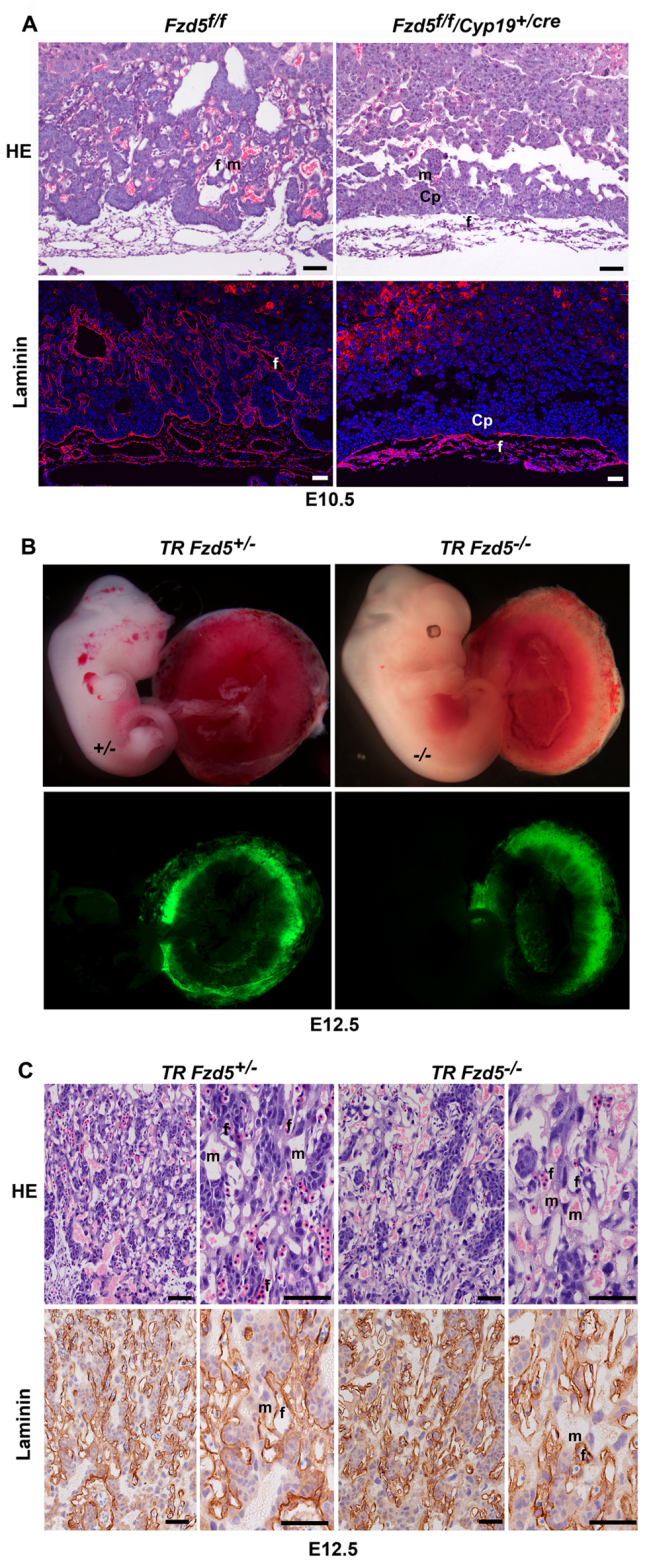
Trophoblast-expressed *Fzd5* is essential for chorioallantoic morphogenesis. (A) HE and laminin staining of E10.5 placentas with Fzd5 deleted specifically in placental trophoblast cells. Note that Trophoblast-specific deletion of *Fzd5* blocked branching morphogenesis in the chorion when analyzed at E10.5. Cy3-labeled laminin in red, Hoechst 33342 labeled nuclei in blue. (B) E12.5 embryos and placentas generated by aggregating wild-type tetraploid *Egfp* ^+/−^ embryos with diploid control or *Fzd5*-null embryos. Note that the GFP-expressing tetraploid cells contributed exclusively to the trophoblast cells of the placenta and the development of diploid *Fzd5*-null embryos was rescued. (C) HE and laminin staining of E12.5 reconstituted placentas generated by tetraploid aggregation. Note that maternal-fetal vascular network developed normally after complementation with wild-type tetraploid trophoblast cells. Cp, Chorionic plate; f, fetal vessel; m, maternal blood sinus. Scale bars: 200 µm.

Since the development of chorionic villi involves both the chorionic trophoblasts and the blood vessels from the allantois, both of which express *Fzd5*, to ascertain the relative contribution of chorionic versus allantoic *Fzd5* in branching morphogenesis, we established a *Cyp19-Cre* ^+/−^ */Fzd5^loxp/loxp^* mouse line by intercrossing *Fzd5^loxp/loxp^* mice with hemizygous *Cyp19-Cre* mice [17],[18] to achieve conditional deletion of the *Fzd5* gene in the trophoblast cells. *Fzd5* gene can be selectively deleted only in the trophoblast cells, while no depletion of *Fzd5* in embryonic tissues (fetal endothelial cells) and yolk sacs was observed (Figure S3A and B). Upon conditional deletion of *Fzd5* in trophoblast cells, retarded fetal growth and blocked branching morphogenesis was observed, similar to that observed in Fzd5-null mutants (Figure 2A and Figure S3C and D). Laminin-stained and Fzd5-intact fetal blood vessels failed to penetrate into the trophoblast-specific *Fzd5*-null chorion, unlike the well-interdigitated maternal-fetal interface in the control (*Fzd5^loxp/loxp^*) chorioallantoic plate (Figure 2A). These data suggest that trophoblast-expressed *Fzd5* is essential for the normal labyrinth development.

On the basis of this finding, we surmised that the placental defects in *Fzd5*-null conceptus would be corrected regardless of the genotype of embryo proper upon complementation with wild-type 4N trophoblasts via tetraploid aggregation assay. Indeed, when diploid *Fzd5*-null embryos were aggregated with wild-type tetraploid *Egfp* ^+/−^ embryos and analyzed at E12.5, the GFP-expressing cells contributed exclusively to the trophoblast cells of the placenta and endoderm of the yolk sac (Figure S4), rescuing the development of diploid *Fzd5* ^−/−^ embryos (Figure 2B). By histological and laminin immunostaining analysis, we further observed that the labyrinth, and its maternal-fetal vascular network developed normally in the tetraploid *Egfp* ^+/−^/*Fzd5* ^−/−^ conceptuses (Figure 2C). These findings highlight the requirement for trophoblast-expressed *Fzd5* during labyrinth development.

### Fzd5 and Gcm1 Are Regulated Reciprocally During the Initiation of Chorionic Branching

**Figure 3:**
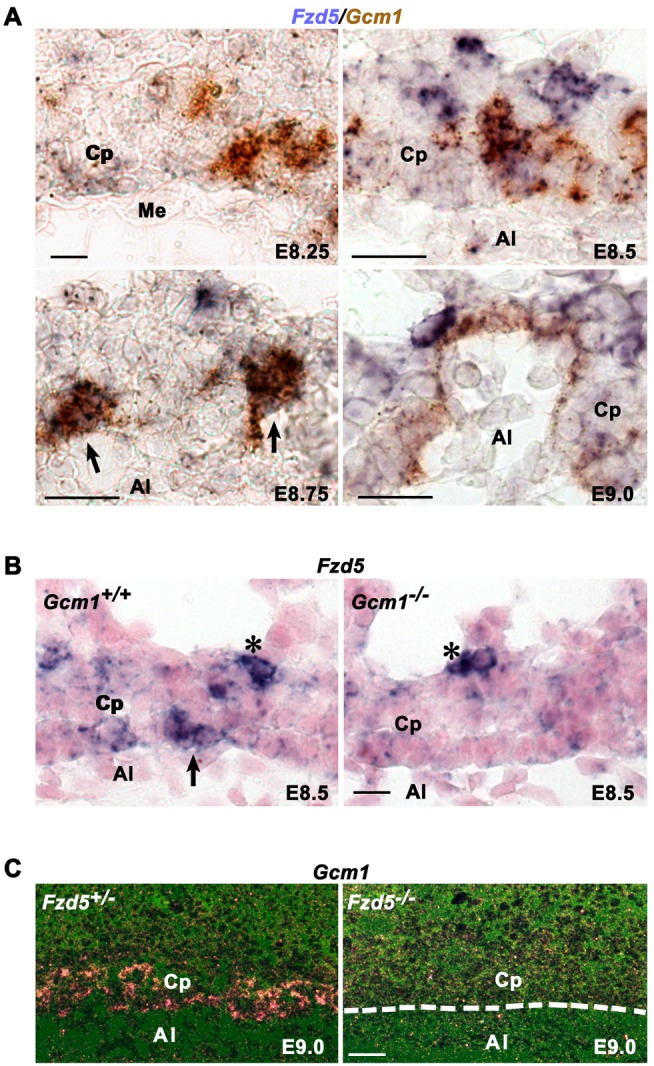
*Fzd5* and *Gcm1* are regulated reciprocally during the initiation of chorionic branching. (A) The expression of *Fzd5* and *Gcm1* revealed by double in situ hybridization. *Fzd5* and *Gcm1* mRNAs were co-localized in trophoblast cells at branchpoint sites along the basal surface of the chorionic plate (arrows). (B) In situ hybridization analysis of Fzd5 mRNA in *Gcm1*-null placentas. *Fzd5* expression was decreased or lack at the base of the chorionic plate in *Gcm1*-null mutant placentas, while *Fzd5* expression at the apical region of the chorion appeared normal (asterisk). (C) *Gcm1* expression detected by in situ hybridization in Fzd5-deficient placentas. *Gcm1* expression was almost diminished in the absence of *Fzd5*. Al, allantois; Cp, Chorionic plate; Me, mesothelium. Scale bars: 200 µm.

It is known that *Gcm1* is expressed in small clusters of chorion trophoblast cells at the flat chorionic plate stage as early as E7.5 [19]–[21] and known to determine the sites where branching initiates [4]. To search for the underlying molecular basis intrinsic to chorionic defects in *Fzd5* mutants, we speculated that there may be regulatory hierarchy between *Gcm1* and *Fzd5* during chorionic branching initiation. To test this idea, we examined expression patterns of *Gcm1* and *Fzd5* in wild-type, *Gcm1* ^−/−^, and *Fzd5* ^−/−^ placentas. Double in situ hybridization analysis revealed a partially overlapping expression pattern of *Gcm1* and *Fzd5* in wild-type chorions (Figure 3A). Before the chorioallantoic attachment stage, patchy expression of *Gcm1* but not *Fzd5* was detected in chorion trophoblasts (Figure 3A). At E8.75 and E9.0, when branching is initiated at the basal layer of the chorionic plate, the *Gcm1* expression was mainly located at the tips of branchpoint sites and lining the branching folds. Expression of *Fzd5* was difficult to detect and scattered in the chorionic plate before E8.5. However, after chorioallantoic attachment *Fzd5* expression became detectable in the chorion but in two distinct regions—in some scattered cells in the apical region as well as in the basal at the branchpoint sites where *Gcm1* is expressed and branching is initiated between E8.75 and E9.0 (Figure 1A and Figure 3A). In *Gcm1* mutant mice, we found a decrease or lack of *Fzd5* expression in the basal chorion at E8.5, while *Fzd5* expression at the apical region of the chorion appeared normal (Figure 3B). Interestingly, we noted that *Gcm1* expression was almost diminished in *Fzd5* mutants (Figure 3C). These interesting findings suggest that *Gcm1* up-regulates *Fzd5* specifically in chorion trophoblast cells at the sites where branching occurs, while this elevated *Fzd5* expression in turn maintains *Gcm1* expression at the branchpoints.

### Fzd5 Deficiency Derails Branching Morphogenesis in the Chorion by Interfering With Trophoblast Syncytialization, Disassociation of Cell Junctions, and Chorionic Fetal Vessel Infiltration

To better understand the sequence of events that are regulated by *Gcm1-Fzd5* function, we analyzed the molecular regulatory machinery governing the syncytiotrophoblast development, trophoblast cell junction disassociation, and blood vessel development from the allantois in *Fzd5* mutants.

**Figure 4:**
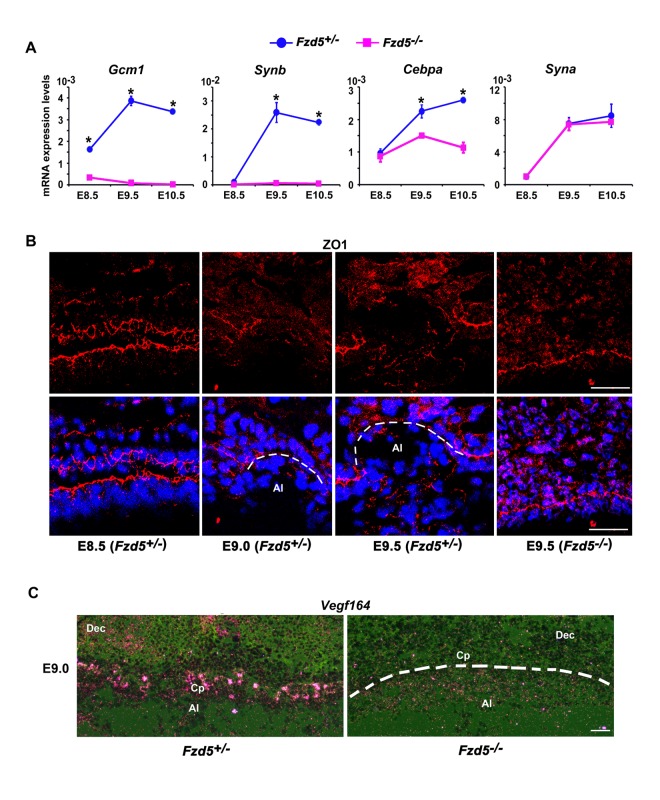
*Fzd5* deficiency derails normal branching morphogenesis by interfering with trophoblast syncytialization, disassociation of cell junction, and chorionic fetal vessel infiltration. (A) Quantitative RT-PCR analysis of marker genes for syncytiotrophoblast cells in control (+/−) and *Fzd5*-null (−/−) placentas. The expression of *Gcm1*, *Synb*, and *Cebpa*, markers for Syn-II cells, was decreased in *Fzd5* mutants. However, *Syna*, a marker for Syn-I cells, was not affected in *Fzd5*-deficient placentas. Values are normalized by GAPDH expression level and indicated as mean±SEM. *N* = 3. *P<0.05. Blue balls and purple blocks represent control (+/−) and *Fzd5* null (−/−), respectively. (B) Immunofluorescence staining of ZO-1 in the chorionic plate during branching morphogenesis. ZO-1 proteins were localized to the apical side of the trophoblast cells throughout the base of the chorionic plate at E8.5. Its expression was significantly down-regulated specifically at the branching sites at E9.0–9.5 in controls (+/−) but not in *Fzd5* mutants. Cy3-labeled ZO-1 in red, Hoechst 33342 labeled nuclei in blue. (C) *Vegf_164_* mRNA expression revealed by in situ hybridization showed lower levels in the chorionic plate of *Fzd5* null compared to control placentas. Al, allantois; Cp, Chorionic plate; Dec, decidua. Scale bars: 200 µm.

In mice, there are two layers of syncytiotrophoblast cells, SynT-I and -II, that lie between the fetal vessels and maternal blood sinuses in the labyrinth, with Syn-I cells lying closest to the maternal blood sinuses and with SynT-II cells lying closest to fetal endothelial cells. In *Fzd5* mutants, in addition to down-regulation of *Gcm1* expression in the chorionic plate at E8.5–10.5 (Figure 4A), expression of Gcm1 target genes syncytin b (*Synb*) and CCAAT/enhancer binding protein α (*Cebpa*), which are all localized to SynT-II cells [21]–[23], was significantly down-regulated (Figure 4A). Moreover, basal chorionic trophoblast cells did not fuse to form syncytiotrophoblast layer II and a functional labyrinth layer failed to form in Fzd5 mutants (Figure S5A and B). By contrast, expression of *Syna*, a marker for *Gcm1*-negative Syn-I cells [21]–[23], was apparently normal in *Fzd5* mutants (Figure 4A). These results suggest that *Gcm1*-directed trophoblast differentiation and syncytialization is greatly hampered in *Fzd5* mutants

Observations of dynamic morphological changes in chorion trophoblast cells at branchpoint (Figure 1C) prompted us to further explore the cellular events that occur during the initiation of branching morphogenesis. Trophoblast cells at the base of chorionic plate are epithelial-like cells, which are linked to each other by cell adhesions, including tight junctions (Figure S6A and B). Dissociation of tight junctions is required for epithelial cells to be transformed to mesenchymal cells [24]. Therefore, we explored the status of the tight junction protein zonula occluden 1 (ZO-1) in the developing chorion by immunofluorescence staining. ZO-1 proteins were localized to the apical side of the trophoblast cells throughout the base of the flat control chorionic plate at E8.5 right before the initiation of branching morphogenesis (Figure 4B), whereas its expression was significantly down-regulated specifically at the branching sites at later times when branching began. By contrast, we noted with interest that ZO-1 expression was sustained in chorion trophoblast cells of *Fzd5* mutants even at E9.5 (Figure 4B). In addition, claudin 4 and 7 underwent similar changes to that of ZO-1 upon Fzd5 deletion (Figure S6A and B). These observations suggested that *Fzd5/Gcm1* is essential for cell junction disassociation, an early step during chorionic branching morphogenesis.

Coincident with chorionic branching in wild-type placentas, the villi are immediately filled with vessels from the allantois. It is conceivable that the invasion of fetal vessels from the allantois into the space created by chorionic branching may be attracted and facilitated by vascular endothelial growth factor (VEGF) [25]. Expression of *Vegf_164_* mRNA was readily detectable in chorion trophoblast cells at E9.0 in control placentas. However, *Vegf_164_* mRNA expression was extremely low in *Fzd5* mutants (Figure 4C), suggesting that fetal vessel infiltration is impaired by Fzd5 mutation.

### Fzd5 Is Essential for Canonical Wnt Activation in Trophoblasts at the Branching Site

**Figure 5:**
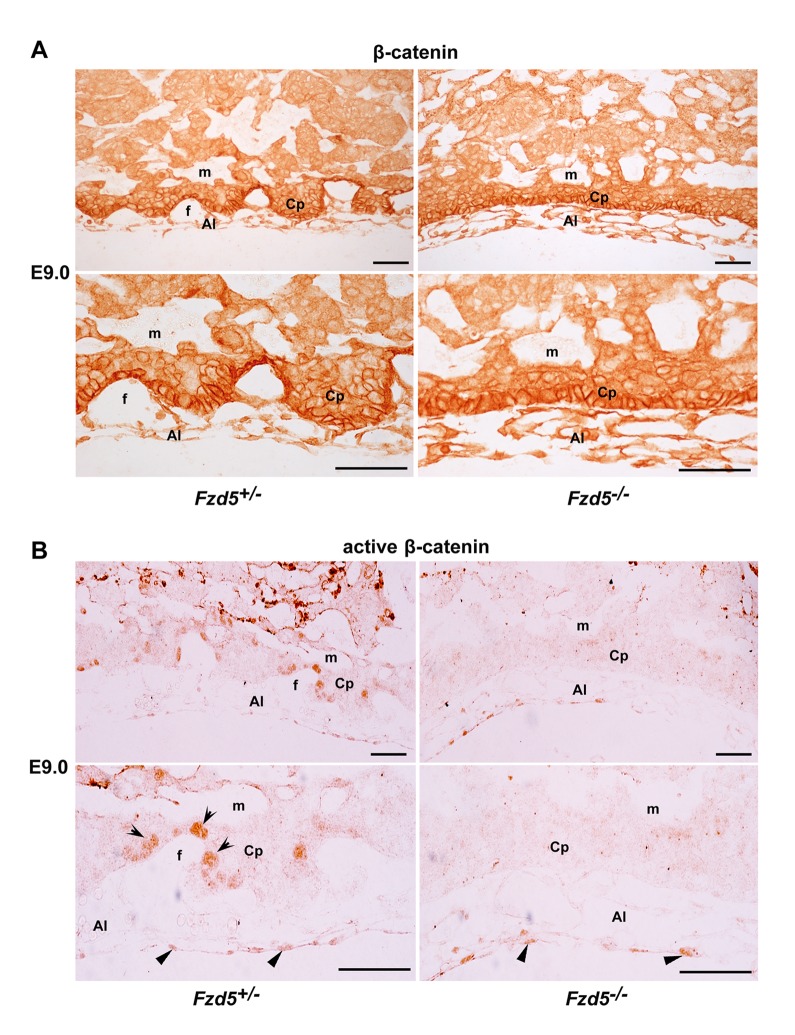
Nuclear localization of active β-catenin is detected in trophoblasts specifically at the branching sites. (A and B) Immunohistochemical localization of β-catenin and active β-catenin in E9.0 placentas. Note while active β-catenin was detected in the nuclei of trophoblast cells lining the branching folds (arrows) in the controls (+/−), it was absent in Fzd5-null (−/−) chorionic trophoblasts. Moreover, nuclear localization of active β-catenin was detected in both the control and Fzd5-null allantois (arrowhead). Al, allantois; Cp, Chorionic plate; f, fetal vessel; m, maternal blood sinus. Scale bars: 200 µm.

Since *Fzd5* deficiency leads to aberrant expression of Gcm1, ZO-1, and VEGF in the chorionic plate and all these genes are known to be regulated by canonical Wnt pathway in other systems [9],[26],[27], we speculated that Fzd5 may mediate the canonical Wnt activation in trophoblast cells during branching morphogenesis. To test this hypothesis, we performed immunohistochemistry to localize β-catenin and active β-catenin in the E9.0 placentas. While the expression of β-catenin was detected in trophoblast cells in both the control and *Fzd5*-null chorionic plate (Figure 5A), nuclear accumulation of active β-catenin was only detected in chorionic trophoblast cells lining branching folds in control placentas, but not in trophoblast cells of Fzd5-null placentas. Moreover, active β-catenin was also detected in the allantois, and its expression was not affected by *Fzd5* deletion (Figure 5B), reinforcing the notion that trophoblast-expressed Fzd5 is essential for normal branching morphogenesis. Nonetheless, this finding provides a new line of evidence suggesting that Fzd5-mediated canonical Wnt pathway plays a role during chorioallantoic development.

### Canonical Wnt2-Fzd5 Signaling Is Essential for Gcm1 Expression During Trophoblast Cell Differentiation in Culture

**Figure 6:**
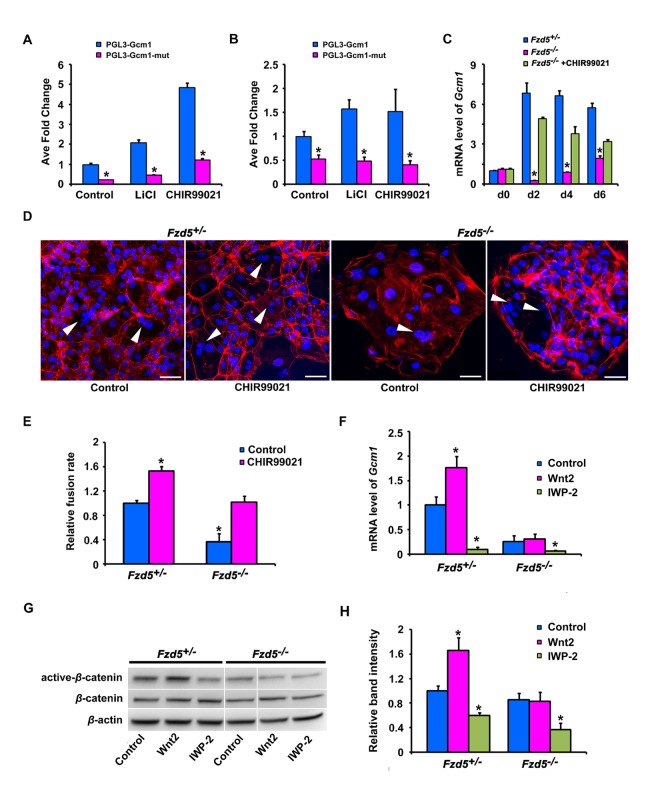
Canonical Wnt2-Fzd5 signaling regulates *Gcm1* expression during trophoblast cell differentiation in culture. (A and B) The activity of Gcm1 promoter when activated by Canonical Wnt pathway. Note that *Gcm1* promoter activity could be regulated by canonical Wnt pathway both in transfected 293T cells (A) and mouse TS cells (B). (C) Gcm1 expression revealed by quantitative RT-PCR in control and Fzd5-null TS cells, with or without CHIR99021 at 0, 2, 4, or 6 d of differentiation under differentiation conditions. CHIR99021, an activator of canonical Wnt pathway, largely restored *Gcm1* expression in *Fzd5* mutant TS cells. Values are normalized by GAPDH expression level and indicated as mean±SEM. *N* = 3. *P<0.05. Blue bars, purple bars, and green bars represent control (+/−), *Fzd5* null (−/−), and *Fzd5* null (−/−) added CHIR99021, respectively. (D) The state of trophoblast syncytialization after treatment with CHIR99021. Trophoblast syncytialization was facilitated by CHIR99021 in both control and *Fzd5*-null TS cells in culture. Syncytiotrophoblast cells were indicated by white arrowhead. Alexa Fluor 555 Phalloidin labeled F-actin in red and Hoechst 33342 labeled nuclei in blue. (E) Quantification of trophoblast syncytialization in control and Fzd5-null TS cells, with or without CHIR99021 at 6 d of differentiation. Cell fusion rate is N/T: N is the number of syncytiotrophoblast cells, and T is the total number of nuclei counted. The results are from three independent experiments; for each, 500–700 cells were counted. (F–H) Overexpression of *Wnt2* in control but not *Fzd5* mutant TS cells elevated *Gcm1* expression (F) and intracellular β-catenin (active β-catenin) accumulation (G and H). However, adding of IWP-2, a small-molecule inhibitor interfering with the ability of cells to produce active Wnt proteins, reduced the levels of *Gcm1* expression and active-β-catenin in both control and *Fzd5*-null TS cells (F–H). Values are normalized by GAPDH expression level and indicated as mean±SEM. *N* = 3. *P<0.05. Blue bars and purple bars represent control (+/−) and *Fzd5* null (−/−), respectively. Scale bars: 200 µm.

Since recent evidence shows that *Gcm1* can be regulated by canonical Wnt pathway in human BeWo choriocarcinoma cells [9], we tested whether *Fzd5*-mediated canonical Wnt signaling would directly regulate *Gcm1* expression during trophoblast differentiation and syncytialization in mice. The PGL3-Gcm1 constructs, which contain binding sites for LEF/TCF, were transfected into HEK293T cells and mouse trophoblast stem (TS) cells. One binding motif (CTTTGTA: −3,661 bp) in the promoter region of *Gcm1* was found to be activated by LiCl and CHIR99021, activators of canonical Wnt pathway (Figure 6A and B and Figure S7). Quantitative RT-PCR analysis further revealed that *Gcm1* expression was dramatically decreased in *Fzd5* ^−/−^ TS cells (Figure 6C), as well as the extent of trophoblast syncytialization (Figure 6D and E), whereas CHIR99021, which bypasses Frizzled receptors [28], could largely restore the *Gcm1* expression and trophoblast syncytialization in *Fzd5* ^−/−^ TS cells (Figure 6C–E). These results indicate that Fzd5-mediated canonical Wnt signaling is essential for normal *Gcm1* expression during trophoblast cell differentiation. However, a question remains as to which Wnt ligand(s) can signal through *Fzd5* receptor during placental development.

Since a reciprocal interaction between allantoic mesoderm and chorionic trophoblast is critical for branching morphogenesis [13]–[15], we surmised that Wnt genes expressed in the allantois would contribute to Fzd5 activation during the initiation of branching morphogenesis. In assessing potential candidates, we performed in situ hybridization analysis of Wnt2, Wnt5a, and Wnt7b gene expression in the developing placentas. We observed that *Wnt2* mRNA was expressed in the allantois before chorioallantoic attachment and to the endothelial cells of the fetal blood vessels at later stages after chorioallantoic attachment. While Wnt7b was localized to the base of the chorion plate, Wnt5a was detected in both the chorion and allantois (Figure S8). Since the trophoblast cells in the chorion begin to differentiate until the attachment of allantois, we surmised that Wnt2 may be a prospective ligand for Fzd5 to trigger trophoblast differentiation in the chorion. To test this hypothesis, we overexpressed Wnt2 in TS cells. Overexpression of Wnt2 significantly upregulated *Gcm1* expression (Figure 6F) in control (*Fzd5* ^+/−^) TS cells, but not in *Fzd5* ^−/−^ trophoblast cells, accompanied by intracellular β-catenin (active β-catenin) accumulation (Figure 6G and H). However, adding of IWP-2, a small-molecule inhibitor interfering with the ability of cells to produce active Wnt proteins [29]–[32], reduced the levels of *Gcm1* expression and active-β-catenin significantly in both control and *Fzd5*-null TS cells (Figure 6G and H), suggesting that Wnt2 is at least one potentially important ligand directing the normal trophoblast differentiation in the chorion during placental development.

### FZD5-GCM1 Signaling Axis Is Operative During Human Trophoblast Syncytialization

**Figure 7:**
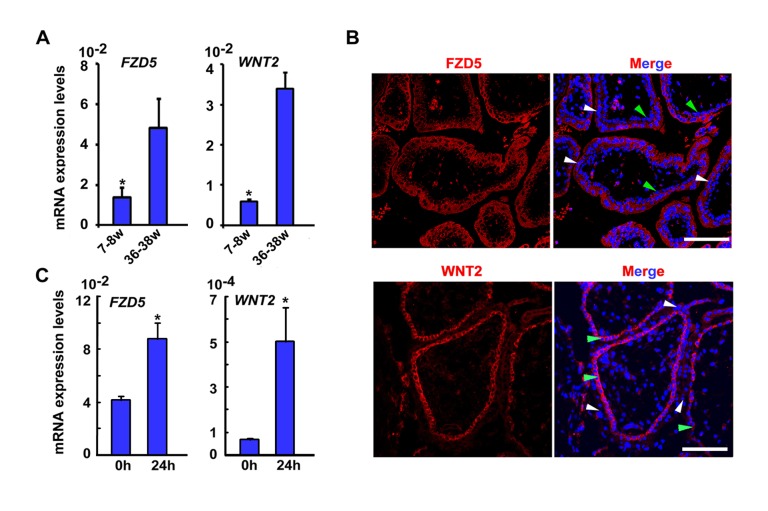
FZD5 and WNT2 are expressed during trophoblast syncytialization in human. (A) Expression of *FZD5* and *WNT2* in human placental villi at 7–8 wk and 36–38 wk of gestation by quantitative RT-PCR. A total of three chorionic villi at weeks 7 and 8 and three placenta specimens at full term (36–38 wk) were enrolled in this study. Values are normalized by GAPDH expression level and indicated as mean±SEM. *N* = 3. *P<0.05. (B) Expression of *FZD5* and *WNT2* in human placental villi detected by immunofluorescence staining. FZD5 was detected in both syncytiotrophoblast cells (white arrowheads) and cytotrophoblast cells (green arrowheads), with more apparent expression in syncytiotrophoblast cells. WNT2 was detected specifically in cytotrophoblast cells. Cy3-labeled FZD5 and WNT2 in red, hoechst33342 labeled nuclei in blue. (C) Expression of *FZD5* and *WNT2* detected by quantitative RT-PCR during spontaneous fusion of isolated primary cytotrophoblast cells. *FZD5* and *WNT2* expression was induced in primary cytotrophoblast cells cultured for 24 h undergoing spontaneous fusion. Values are normalized by GAPDH expression level and indicated as mean±SEM. *N* = 3. *P<0.05.

**Figure 8:**
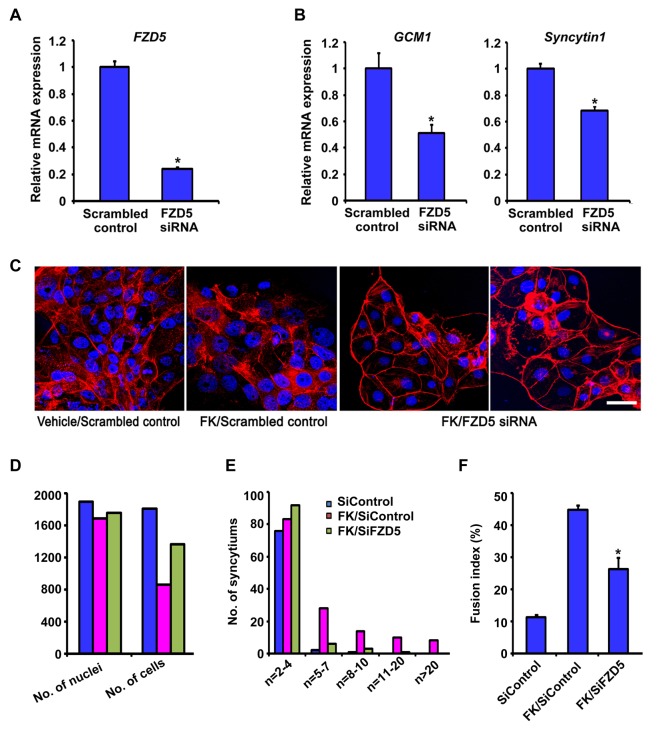
The *FZD5-GCM1* signaling axis is operative during human trophoblast syncytialization. (A) Efficiency of *FZD5* mRNA knockdown by SiRNA in BeWo cells revealed by quantitative PR-PCR. (B) Expression of *GCM1* and *Syncytin1* detected by quantitative RT-PCR was down-regulated in BeWo cells upon *FZD5* knockdown. Values are normalized by GAPDH expression level and indicated as mean±SEM. *N* = 3. *P<0.05. (C) *FZD5* mRNA knockdown decreased the extent of cell fusion of BeWo cells after 48 h of forskolin (FK) treatment revealed by immunostaining. Cy3-labeled β–catenin in red, Hoechst 33342 labeled nuclei in blue. Scale bars: 200 µm. (D) Quantification of the total number of cells or syncytium and their nuclei. (E) Quantification of the number of nuclei per cell or syncytium. The *y*-axis is in a logarithmic scale. (F) Fusion index revealed by quantifying the events of cell–cell fusion. Fusion index is [(N−S)/T]×100%: N is the number of nuclei in the syncytia, S is the number of syncytia, and T is the total number of nuclei counted. *N* = 3.

As in mice, GCM1 regulates trophoblast syncytialization in humans [33], and so we examined if WNT2-FZD5 signaling was also involved. *FZD5* mRNA was detected in both cytotrophoblasts and syncytiotrophoblasts in human placental villi (Figure 7A and B), and its expression was up-regulated in cultured primary cytotrophoblast cells undergoing spontaneous fusion into syncytiotropblasts (Figure 7C). Moreover, *WNT2* was primarily expressed in cytotrophoblast cells of the human villous (Figure 7A and B) and its expression was increased during spontaneous fusion of primary cytotrophoblast cells (Figure 7C). To determine whether *FZD5* would regulate trophoblast syncytialization in humans, we employed siRNA against different sequences within the *FZD5* mRNA in human BeWo cells. Upon down-regulation of *FZD5* expression after introducing siRNA (Figure 8A), expression of *GCM1* was down-regulated (Figure 8B). Moreover, FZD5 siRNA also reduced the expression of *Syncytin 1* (Figure 8B), a downstream target gene of *GCM1*, which mediates the fusion of cytotrophoblast cells into syncytiotrophoblast cells [34],[35]. Since up-regulated *GCM1* and *Syncytin1* are required for forskolin (FK)-induced fusion of BeWo cells [33], we subsequently examined whether silencing of FZD5-mediated signaling would hamper FK-induced cell–cell fusion of BeWo cells. Indeed, we noted that FZD5 siRNA largely abolished the cell fusion events in FK-treated BeWo cells (Figure 8C–F). These findings suggest that the role of FZD5-GCM1 signaling in regulating trophoblast syncytialization is conserved from mouse to human.

## Discussion

**Figure 9:**
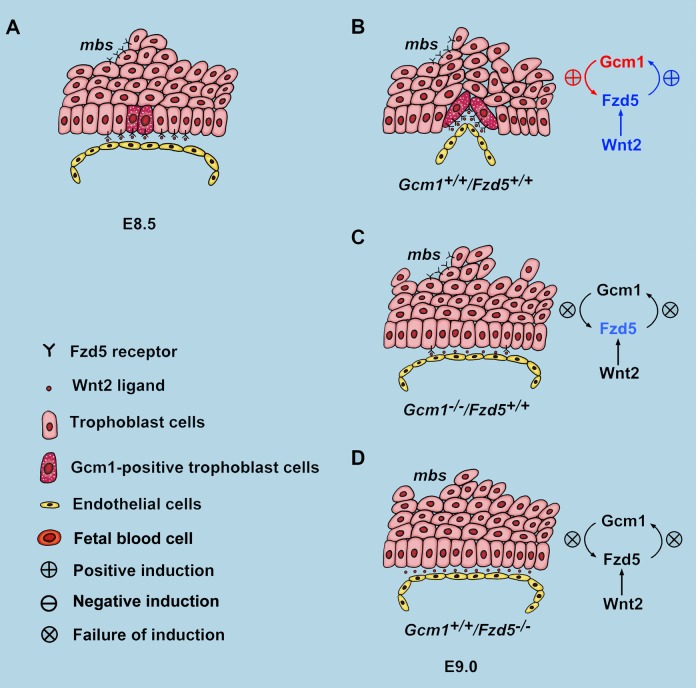
Diagram illustrating the regulatory hierarchy between *Gcm1* and *Fzd5* in chorionic trophoblasts during branching initiation in mice. (A) At E8.5, *Gcm1* was expressed specifically at trophoblast cells where branching is to be initiated. However, the expression of Fzd5 was low and across the base of the chorionic plate. (B) By E9.0, *Gcm1* and *Fzd5* expression was up-regulated through reciprocal induction at chorion branchpoints. (C and D) Failure of branching morphogenesis in *Gcm1* and *Fzd5* mutants.

The labyrinth layer of the placenta is the only site for exchange of nutrients, gases, and wastes between the maternal and fetal circulations from midgestation to term. Chorioallantoic attachment is the first step during labyrinth development, but soon thereafter, primary villi begin to develop at specific sites along the basal surface of the chorion that quickly become lined by fetoplacental blood vessels from the allantois. Defects in these processes are one of the most common causes of midgestation embryonic lethality. However, much remains unclear about the mechanisms. We provide here genetic, molecular, pharmacological, and physiological evidence that an amplifying feedback loop between Gcm1 and Fzd5 is essential for normal initiation of branching and trophoblast differentiation in the chorion of mice (Figure 9). Moreover, our studies reveal that this signaling axis is also functional in the human placenta.

Previous studies have proposed that the trophoblast cells at the branching sites within the chorion express *Gcm1* and that changes in cell shape—thinning and elongation—are involved in driving the branching morphogenesis [16]. Deletion of *Gcm1* in mice leads to a complete block to branching at the chorioallantoic interface. We observe a similar phenotype of impaired chorionic branching in *Fzd5* mutant mice, even those lacking only trophoblast-expressed *Fzd5*. *Fzd5* is expressed in clusters of cells in the apical as well as the basal chorion, though only the latter sites correlate with branching morphogenesis and overlap with *Gcm1*. *Gcm1* expression precedes that of *Fzd5* in the chorion and it was of great interest to note that *Gcm1* deficiency remarkably attenuates *Fzd5* expression at the site of branchpoint initiation in the basal chorion. This implies that Gcm1 regulates the onset of Fzd5 expression in the basal chorion, but we assume that this is not due to direct transcriptional activation by Gcm1 since the regulatory elements of *Fzd5* gene have no *Gcm1* binding motifs (unpublished observation) [35]. Therefore, it is possible that the regulation of Fzd5 by Gcm1 was mediated by other indirect ways.

While *Gcm1* precedes *Fzd5* expression, *Fzd5* is in turn essential for the maintenance of *Gcm1* expression at the selected branching site. *Fzd5* mutant chorions had diminished *Gcm1* expression and impaired nuclear localization of β-catenin at E9.0 and beyond. Employing TS cells, we found that Fzd5 through nuclear β-catenin signaling directly regulates *Gcm1* expression during trophoblast differentiation. Our findings are consistent with recent studies about two important members of canonical Wnt pathway, R-spondin3 and *Bcl9l*. Mutations in genes encoding R-spondin3, a protein that promotes the Wnt-β-catenin signaling pathway, result in failure of chorionic branching and reduced Gcm1 expression [8]. *Bcl9l* is an essential intracellular member of Wnt pathway and functions as an adaptor linking β-catenin and Pygopus. Its deficiency leads to defective branching initiation and impaired differentiation of trophoblast cells in the chorion into syncytiotrophoblast layer II (SynT-II) cells [9]. In general, these observations further testify the important role of Fzd5-mediated canonical Wnt pathway on Gcm1 regulation during placental development.

Our studies add to the understanding of the upstream events that initiate where chorionic branching will occur. However, the cellular events downstream of Gcm1 and Fzd5 that drive chorion trophoblast differentiation and morphogenesis have not previously been well documented. In mice, trophoblast cells at the basal layer of the chorionic plate are aligned and tightly adherent to each other, similar to other polarized epithelia. This epithelium integrity is maintained by cell junctions, particularly the tight junctions at the apical side of epithelium. With the initiation of branching, the trophoblast cells that express *Gcm1* at the branching sites become thin and elongated, and disassociated similar to an epithelial to mesenchymal transition. However, a reduction of E-cadherin and up-regulation of vimentin has not been observed in chorion trophoblasts at the branching sites (unpublished observation). By contrast, the expression of ZO-1 and claudin4 and 7, important components of tight junctions, is dramatically reduced or diminished at the branching sites in the control chorion, whereas this down-regulation does not occur in *Fzd5* ^−/−^ mutants. Moreover, ZO-1 can be down-regulated by Wnt-β-catenin signaling in human colorectal carcinomas [26]. This suggests that Fzd5 is essential for down-regulation of ZO-1 and claudins expression during branching initiation.

Any defect in branching morphogenesis of the chorion results in a small labyrinth layer, thus limiting the surface area for nutrient transfer as well as the extent to which fetal blood vessels can grow into the placenta and come into proximity to the maternal blood spaces. To the untrained observer and at a superficial level, mutants with small labyrinth layers due to chorion branching defects may appear to be undervascularized, but it is critical to distinguish the actual events and determine whether the volume of the fetal vascular network in the labyrinth is simply small compared to wild-type because it is proportionally limited by a reduced villous volume, as is true in many cases [2], or whether it is disproportionately reduced. It is worthy of further investigation to determine if there are vascular defects in *Fzd5* mutants. Previous studies on *Wnt2* mutants described an impaired fetal vascular network in the labyrinth [10], but previous descriptions of placental vascular defects in *Fzd5* mutants [11] cannot be confirmed based on our findings of primary chorion branching defects. Whether Wnt2-Fzd5 signaling is essential for regulating the subsequent vascularization of villi after the primary villous branching occurs will require further studies. However, we provide evidence here that trophoblast-expressed Fzd5 is essential for *Vegf* expression in the chorionic plate, coincident with the sites of primary villous branching that are filled in by vessels from the allantois. VEGF can function as a chemoattractant as well as a growth factor to promote vessel growth [25] and can be strongly up-regulated by Wnt signaling during tumorigenesis [27]. Aberrant expression of *Vegf* has also been shown to be associated with severely impaired labyrinth morphogenesis, although the branching can be initiated in *Lkb1* and *Tfeb* mutant mice [36],[37]. These findings suggest that Gcm1/Fzd5 signaling initiates not just differentiation and branching morphogenesis in the chorion trophoblast but that the trophoblast may in turn regulate vascularization of the labyrinth.

In summary, we provide direct genetic evidence of an amplifying feedback loop of Gcm1-Fzd5 signaling in the chorion and propose three main conclusions: (1) *Gcm1*, first expressed in chorion trophoblast cells and further upregulated by canonical *Fzd5* signaling, determines the branching sites and differentiation into syncytiotrophoblasts; (2) the initial events in chorion trophoblast morphogenesis include trophoblast cell cycle exit and downregulation of ZO-1 expression, inducing the disassociation of tight junctions at the base of the chorionic plate for branching initiation; and (3) Wnt-Fzd5 signaling also up-regulates *Vegf* expression in the chorion and may in turn promote vascularization of the primary villi in the labyrinth. Besides shedding light on the fundamental mechanisms of branching morphogenesis during placental development, the finding has high clinical relevance, since Gcm1-Fzd5 signaling cascade is operative during human trophoblast differentiation and its aberrant regulation is often associated with trophoblast-related diseases, such as preeclampsia [38]–[40].

## Materials and Methods

### Animals and Tissue Collection

*Fzd5^loxp/loxp^* mice, *Gcm1*-null mice, and *Cyp19-Cre* transgenic mice were generated as previously described in Drs. Hans Clevers, Gustavo Leone, and James C. Cross's groups, respectively [4],[12],[17],[18]. Enhanced green fluorescent protein (*Egfp*), Rosa26^loxp/loxp^, and *Zp3-Cre* transgenic mice were obtained from Jackson Laboratory. Mice were housed in Institutional Animal Care Facility according to institutional guidelines for laboratory animals. Females were mated with fertile males of the same strain to induce pregnancy (E0.5, vaginal plug). Conceptuses for RNA extraction and histology were dissected from uteri from E8.0 to E12.5 as previously described [41],[42]. For double in situ hybridization, whole implantation sites or dissected placentas were fixed with 4% paraformaldehyde (PFA) in phosphate-buffered saline (PBS) at 4°C overnight. After PBS washes, tissues were immersed in 10% and 25% sucrose in PBS, and then embedded in the Tissue-Tek OCT compound and frozen with dry ice-cooled ethanol. All tissues used for other analysis were fresh-frozen or fixed in 10% neutral buffer formalin (NBF).

Tissues of human chorionic villi at gestational weeks 7 and 8 from pregnant women undergoing therapeutic termination of pregnancy, and human placental tissues at full term (36–38 wk) were obtained from Xuan-Wu Hospital and the Department of Obstetrics and Gynecology, Peking University Third Hospital in Beijing, China. Termination of pregnancies at weeks 7 and 8 and virginal deliveries at full term were conducted as per usual clinical practice, with no specific procedures relevant to this research study. The study was approved by the Research Ethic Committee in the Institute of Zoology and that in Xuan-Wu Hospital and Peking University Third Hospital. All the pregnant women provided the written informed consent of using the placenta tissues for research work regarding the expression of Wnt signaling before they denoted the placentas. A total of three chorionic villi at weeks 7 and 8 and three placenta specimens at full term (36–38 wk) were enrolled in this study. All placental tissues were washed with ice-cold PBS and fresh-frozen or fixed in 4% PFA for further analysis.

### Histological Analysis and Immunostaining

For hematoxylin and eosin staining, isolated implantation sites or whole dissected placentas were fixed in 10% NBF, dehydrated and embedded in paraffin wax, and cut into 5-µm sections. For semithin and ultrathin resin histology, implantation sites were fixed in 2% glutaraldehyde and embedded in JB-4 epoxy resin according to the manufacturer's instructions (Electron Microscopy Sciences). Sections (semithin, 2 µm; ultrathin, 100 nm) were then cut using glass knives on a Leica RM2265 microtome. Semithin sections were stained with Toluidine blue (Amresco) and ultrathin sections were contrasted with uranyl acetate and lead citrate. For immunohistochemistry analysis, antibodies specific to Laminin affinity-isolated antigen-specific antibody (Sigma), cow cytokeratin (Dako), phosphor-histone H3 (Cell Signaling), Ki67 (Epitomics), and active-β-catenin (Millipore) were used in 5-µm thick paraffin embedded sections. A Histostain-SP Kit (Zhongshan Golden Bridge Biotechnology) was used to visualize the antigen. For immunofluorescence, antibodies specific to ZO-1 (Abcam), WNT2 (R&D), FZD5 (Abcam), Claudin4 (Anbo), and Claudin7 (Anbo) and secondary antibodies conjugated with Cy3 dyes (Jackson ImmunoResearch Laboratories) were used. To analyze the cell fusion status, immunolocalization using β-catenin (Abcam) or Alexa Fluor 555 Phalloidin (Life Technology) was performed. Immunofluorescence images were captured in a Zeiss LSM 510 confocal scanning laser microscope.

### In Situ Hybridization

In situ hybridization with isotopes, digoxygenin (DIG), or fluorescein isothiocyanate (FITC)–labeled antisense RNA probes was performed on cryosections as described previously [21],[43]. Sections hybridized with the sense probes served as negative controls.

### Tetraploid Aggregation Assay

Tetraploid aggregation chimeras were generated as described previously [44] with some modifications. Briefly, wild-type tetraploid embryos were generated by electrofusion of two-cell embryos derived from EGFP intercrosses. Fused embryos were cultured overnight in KSOM medium. Diploid eight-cell or morula-stage embryos generated from *Fzd5* ^+/−^ intercrosses were collected on E2.5. Zona pellucidae were removed with acidic Tyrode solution, and each diploid embryo was aggregated with two tetraploid embryos. Aggregated chimeric embryos were allowed to develop to the blastocyst stage and were then transferred into the pseudopregnant uteri of wild-type females. Chimeric embryos were dissected at E12.5. Both the embryo proper and placenta were visualized for GFP under a dissecting microscope and subsequently fixed and stained with hematoxylin-eosin and laminin. The visceral endoderm and embryo tissue was used as a DNA source for genotyping of diploid embryos.

### Real-Time RT-PCR Analysis

Total RNA was isolated from chorionic plate or placenta by using TRIZOL (Invitrogen). One microgram of total RNA was used to synthesize cDNA. Expression levels of different genes were validated by real-time RT-PCR TaqMan analysis using the ABI 7500 sequence detector system according to the manufacturer's instructions (Applied Biosystems). All primers for real-time PCR were listed in Table S1. Assays were performed at least three times with each in duplicate.

### Plasmid Construction

The coding sequence region for *Wnt2* was amplified from mouse placenta and cloned in to PWPI expression vector. The expression of Wnt2 was confirmed by transfecting into HEK293T cells followed by RT-PCR or Western blot. The upstream region of the *Gcm1* gene relative to the transcription start site was generated by PCR (primers listed in Table S1) using placental genomic DNA as template. The amplified fragments were cloned into pGL3-Basic vectors (Promega). Mutations of the LEF/TCF binding sites were achieved by Fast Mutagenesis System (Stratagene).

### Cell Culture, Transfection, Luciferase Assay, and siRNA Knockdown

HEK293T cells were kept in DMEM medium (HyClone) supplemented by 10% serum, 1 mM sodium pyruvate, 2 mM L-glutamine. *Fzd5* ^+/−^ and *Fzd5* ^−/−^ TS cells were derived from E3.5 mouse blastocysts as described previously [45]. Established TS cells were maintained in a proliferative state in media containing 70% embryonic fibroblast-conditioned medium, 30% TS cell medium, FGF4 (25 ng/ml), and heparin (1 µg/ml). For differentiation conditions, TS cell medium was used but without supplementation with bFGF, heparin, and embryonic fibroblast pre-conditioning in the presence of CHIR99021 (Biovision) or not. All constructs were transiently transfected into HEK193T cells and TS cells using Lipofectamine LTX and PLUS reagents (Invitrogen) according to the manufacturer's instructions. pRL-TK, internal control plasmid expressing Renilla (Promega), was co-transfected into the cells to normalize firefly luciferase activity of the reporter plasmids. LiCl (Sigma) and CHIR99021 were added 24 h after transfection and cells were collected after another 24 or 48 h, for HEK293T and TS cells, respectively. Luciferase assay was performed by Dual-Luciferase Reporter System (Promega) according to the manufacturer's instructions. Assays were performed at least three times with each in duplicate.

The human choriocarcinoma BeWo cell line was obtained from American Type Culture Collection and maintained as monolayers at 37°C, 5% CO_2_ with F-12K/DMEM, (1∶1) medium (Gibco) supplemented with 10% fetal bovine serum, and 2 mM glutamine. For RNA interference experiments, 20 nM of siRNAs were reverse transfected into 6.5×10^4^ BeWo cells per 500 µl in 24-well plates (or adjusted proportionally to the plate size) by Lipofectamine RNAiMAX (Invitrogen) according to the manufacturer's instructions. At 24 h after transfection, medium was changed to that containing 50 µM FK or vehicle (dimethyl sulfoxide) and collected at 72 h posttransfection for assay. Sequences for siRNA oligonuclotides are listed in Table S1. Stealth RNAi siRNA Negative Control Hi GC or Med GC was used as control siRNA.

### Cell Fusion Assay

The analysis of cell fusion is according to the procedure described previously [9]. In brief, BeWo cells with siRNA transfected in a 24-well plate were immunostained by standard procedure and then observed at a final magnification of 400×. Six microscopic fields per sample were randomly selected for examination; three independent experiments were performed.

### Statistical Analysis

Statistical analysis was performed with SPSS11.5 program. Comparison of means was performed using the independent-samples *t* test. Data were showed as means ± SEM.

## Supporting Information

Figure S1 Fzd5 expression was detected during early placentation. (A) RT-PCR analysis of Fzd5 expression in allantois, chorion, and yolk sacs. Fzd5 was detected both in E8.0 allantois and chorion, as well as in yolk sacs at later developmental stages. (B) The expression of Fzd5 revealed by in situ hybridization. Fzd5 expression was high at the tips of branchpoints after chorioallantoic attachment (arrow). (C) The expression of Fzd5 was observed by Western blot in E8.5 and E9.5 placentas. (D) Immunostaining of Fzd5 in E9.0 chorionic plate with apparent expression at the branching sites. Cy3-labeled FZD5 in red, Hoechst 33342 labeled nuclei in blue. Al, allantois; Cp, Chorionic plate; f, fetal vessel; m, maternal blood sinus. Scale bars: 200 µm.
(TIF)

Figure S2 Attachment of the chorion and allantois was not affected in *Fzd5* mutants. (A) Quantitative RT-PCR analysis of Itga4 and Vcam1 in the control and Fzd5-null placentas at E8.5. The expression of vascular cell adhesion molecule-1 (VCAM-1) and α4 integrin, which are required for chorioallantoic attachment, were normal with Fzd5 deletion. Values are normalized by GAPDH expression level and indicated as mean±SEM. *N* = 3. *P<0.05. (B) The expression of integrinα4 revealed by immunostaining. The expression of α4 integrin, localized to the base of chorion plate, was not affected in Fzd5-deficient mice. Cy2-labeled integrinα4 in green, Hoechst 33342 labeled nuclei in blue. Scale bars: 200 µm.
(TIF)

Figure S3 Conditional deletion of Fzd5 gene in trophoblasts during placental development in mice. (A and B) The efficiency of conditional deletion of Fzd5 by *Cyp19-Cre* in trophoblast cells was detected by RT-PCR (A) and LacZ staining of placentas from *Rosa26^loxp/loxp^* mice and *Cyp19-Cre* ^+/−^ mice intercross (B). Fzd5 gene could be selectively deleted in the trophoblast cells (arrows), while no depletion of Fzd5 in embryonic tissues (fetal endothelial cells) (arrowheads) and yolk sacs was observed. (C) Whole mount views of E10.5 control (Fzd5^f/f^) and *Fzd5* conditional-deleted (Fzd5^f/f^/Cyp19^+/Cre^) placentas and yolk sacs. Trophoblast specific deletion of Fzd5 led to fetal growth retardation and pale yolk sacs with no blood perfusion. (D) Placental and fetal weight after Fzd5 conditional deletion by Cyp19Cre at E10.5. While the placental weight was not affected by Fzd5 conditional deletion, the fetal weight was reduced significantly (*P*<0.05). (E) PCR of genomic DNA from tetraploid rescued embryos as well as mixed DNA templates showing that wild-type tetraploid cells were excluded from the embryo proper. Numbers above the lanes represent the percentage of wild-type DNA in the wild-type/Fzd5 mutant mixed DNA templates. f, fetal vessel; La, labyrinth; m, maternal blood sinus; Sp, spongiotrophoblast layer; Yc, yolk sac; B, brain; L, lung; H, heart; M, muscle. Scale bars: 200 µm.
(TIF)

Figure S4 Tetraploid trophoblast with EGFP contributed exclusively to the endoderm of the yolk sacs. E12.5 fetus within its yolk sac was with intact placenta generated by tetraploid aggregation after complementation with wild-type tetraploid *Egfp* ^+/−^ embryos. Note that the contribution of tetraploid trophoblast cells with EGFP to the endoderm of the yolk sac (arrowhead).
(TIF)

Figure S5 Impaired labyrinth formation caused by Fzd5 deletion. (A) HE staining of E10.5 control (+/−) and *Fzd5*-null (−/−) placentas. Note that the fetal vessel didn't penetrate into the chorion to interdigitate with the maternal sinuses and a functional labyrinth layer failed to form in the *Fzd5*-null (−/−) placentas. Scale bars: 400 µm. (B) Analysis of chorionic trophoblasts in E10.5 placentas by electron microscopy. While elongation and fusion of chorionic trophoblast cells to form syncytiotrophoblast layer II between the fetal blood vessels and maternal sinus in Control (+/−) placentas, basal chorionic trophoblast cells remain unfused and undifferentiated in *Fzd5*-null (−/−) placentas. f, fetal vessel; m, maternal blood sinus; B-CT, basal chorionic trophoblasts; ec, fetal endothelial cells; fc, fetal blood cells; ms, maternal blood sinus; ST, syncytiotrophoblast cells; stgc, sinusoidal trophoblast giant cells. Scale bars: 5 µm.
(TIF)

Figure S6 Tight junctions revealed by claudins exist between the trophoblast cells at the basal chorionic plate. (A and B) The expression of Claudin4 (A) and Claudin7 (B) at E9.0 chorionic plate was revealed by immunostaining. Note that the Claudin4 and 7 are mainly expressed at the apical side of the trophoblast cells at the base of the chorionic plate, and their expression was reduced or diminished at the branching sites (arrowheads). Al, allantois; Cp, Chorionic plate. Scale bars: 200 µm.
(TIF)

Figure S7 Analysis of LEF/TCF binding sites in the Gcm1 promoter. (A) Map of the Gcm1 promoter, indicating the 5-kb region containing the LEF/TCF binding sites. A fragment containing nucleotides (nt) −4,117 to −3,338 relative to the transcription site (P) is comprised of seven binding sites for LEF/TCF complex. (B) Point mutations of the seven binding sequences revealed that only the fifth sequence was responsive to canonical Wnt pathway agonists, LiCl and CHIR99021 (red in A).
(TIF)

Figure S8 The expression of Wnt2, Wnt5a, and Wnt7b was detected by in situ hybridization during early placentation. Wnt2 was expressed in the allantois before chorioallantoic attachment at E8.0 and was localized to the endothelial cells of the fetal blood vessels at later stages. While Wnt7b was localized to the base of the chorion plate, Wnt5a was detected in both the chorion and allantois. Al, allantois; Ch, Chorion; Dec, decidua; Epc, ectoplacental core; La, labyrinth layer; Sp, spongiotrophoblast layer. Scale bars: 200 µm.
(TIF)

Table S1 Oligonucleotides.
(DOC)

## Abbreviations

Bcl9l: B cell CLL/lymphoma 9-like
Cebpa: CCAAT/enhancer binding protein α
*Egfp*: Enhanced green fluorescent protein
EMFI-CM: embryonic mouse fibroblast cell conditioned medium
FGF: fibroblast growth factor
Fzd5: Frizzled 5
Gcm1: Glial cells missing-1
LiCl: Lithium chloride
pH3: phospho-histone H3
Syna: syncytin a
Synb: syncytin b
SynT: syncytiotrophoblast layer
TGC: trophoblast giant cell
VEGF: vascular endothelial growth factor
ZO-1: zonula occluden 1
