# Supplementary figures and images for: A Positive Feedback Loop Involving Gcm1 and Fzd5 Directs Chorionic Branching Morphogenesis in the Placenta
Source: PLoS Biol. 2013 Apr 16;11(4):e1001536. doi: 10.1371/journal.pbio.1001536 (PMC3627642; doi:10.1371/journal.pbio.1001536)

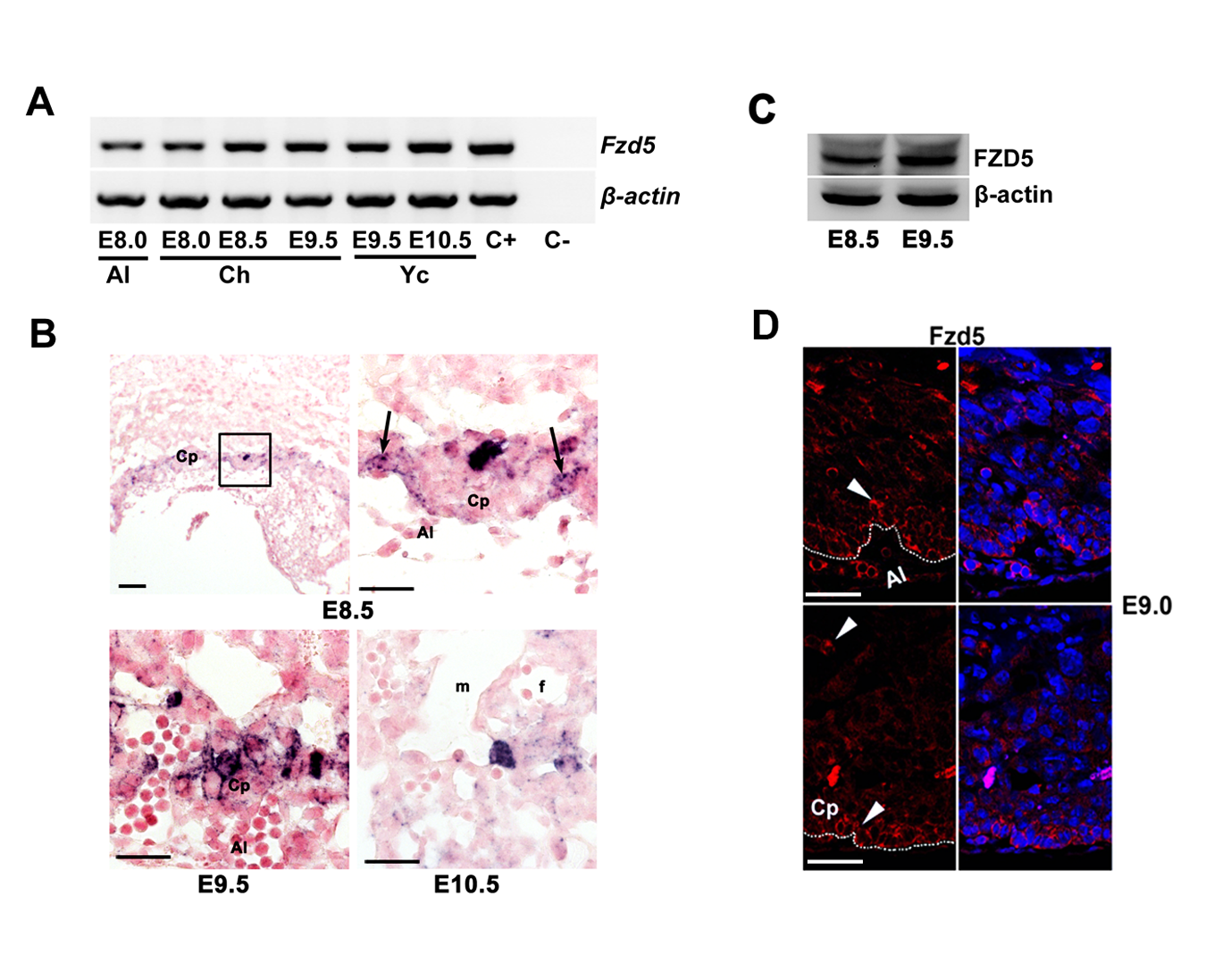

Supplement: Figure S1 — Fzd5 expression was detected during early placentation. (A) RT-PCR analysis of Fzd5 expression in allantois, chorion, and yolk sacs. Fzd5 was detected both in E8.0 allantois and chorion, as well as in yolk sacs at later developmental stages. (B) The expression of Fzd5 revealed by in situ hybridization. Fzd5 expression was high at the tips of branchpoints after chorioallantoic attachment (arrow). (C) The expression of Fzd5 was observed by Western blot in E8.5 and E9.5 placentas. (D) Immunostaining of Fzd5 in E9.0 chorionic plate with apparent expression at the branching sites. Cy3-labeled FZD5 in red, Hoechst 33342 labeled nuclei in blue. Al, allantois; Cp, Chorionic plate; f, fetal vessel; m, maternal blood sinus. Scale bars: 200 µm. (TIF) [file pbio.1001536.s001.tif]

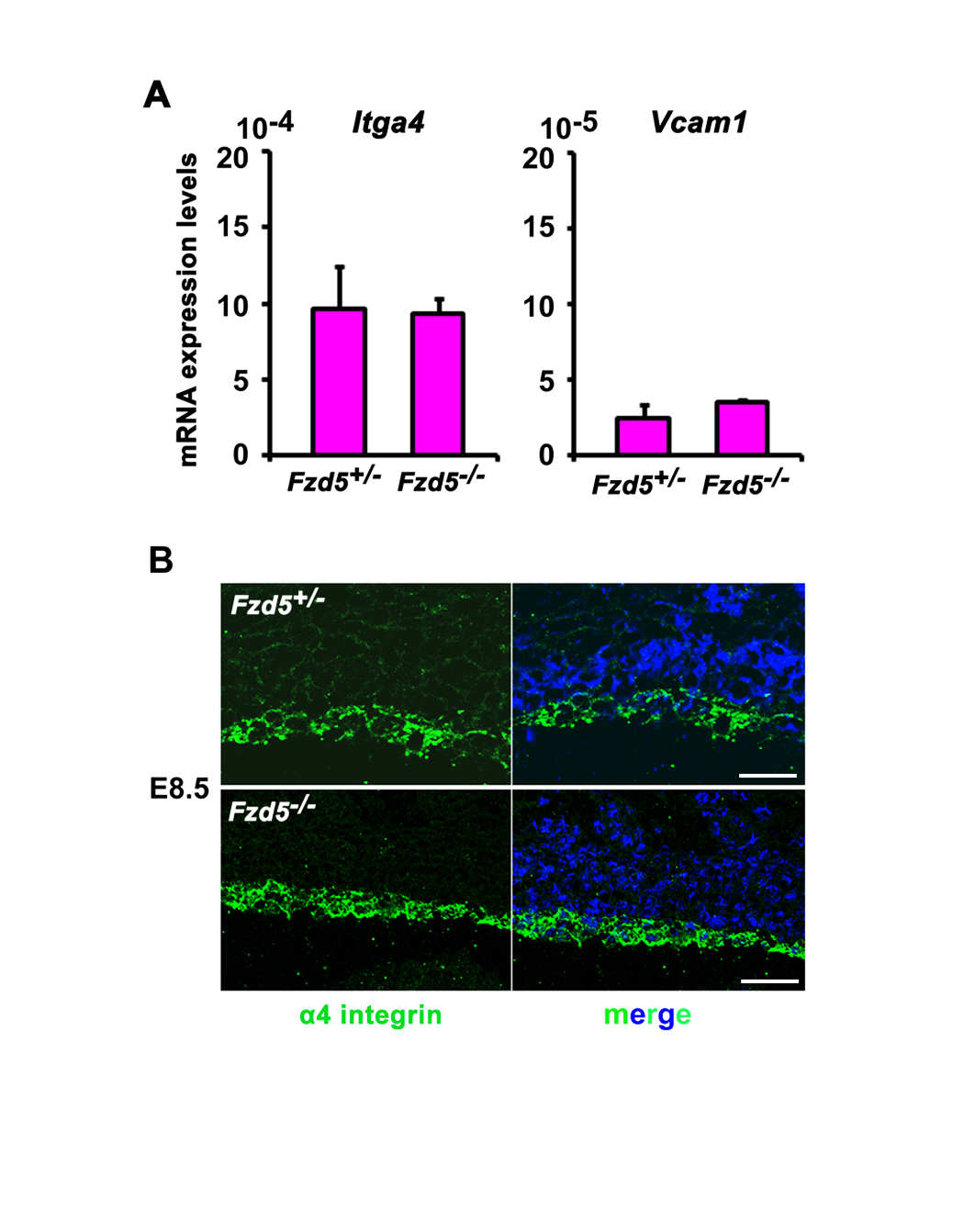

Supplement: Figure S2 — Attachment of the chorion and allantois was not affected in Fzd5 mutants. (A) Quantitative RT-PCR analysis of Itga4 and Vcam1 in the control and Fzd5-null placentas at E8.5. The expression of vascular cell adhesion molecule-1 (VCAM-1) and α4 integrin, which are required for chorioallantoic attachment, were normal with Fzd5 deletion. Values are normalized by GAPDH expression level and indicated as mean±SEM. N = 3. *P<0.05. (B) The expression of integrinα4 revealed by immunostaining. The expression of α4 integrin, localized to the base of chorion plate, was not affected in Fzd5-deficient mice. Cy2-labeled integrinα4 in green, Hoechst 33342 labeled nuclei in blue. Scale bars: 200 µm. (TIF) [file pbio.1001536.s002.tif]

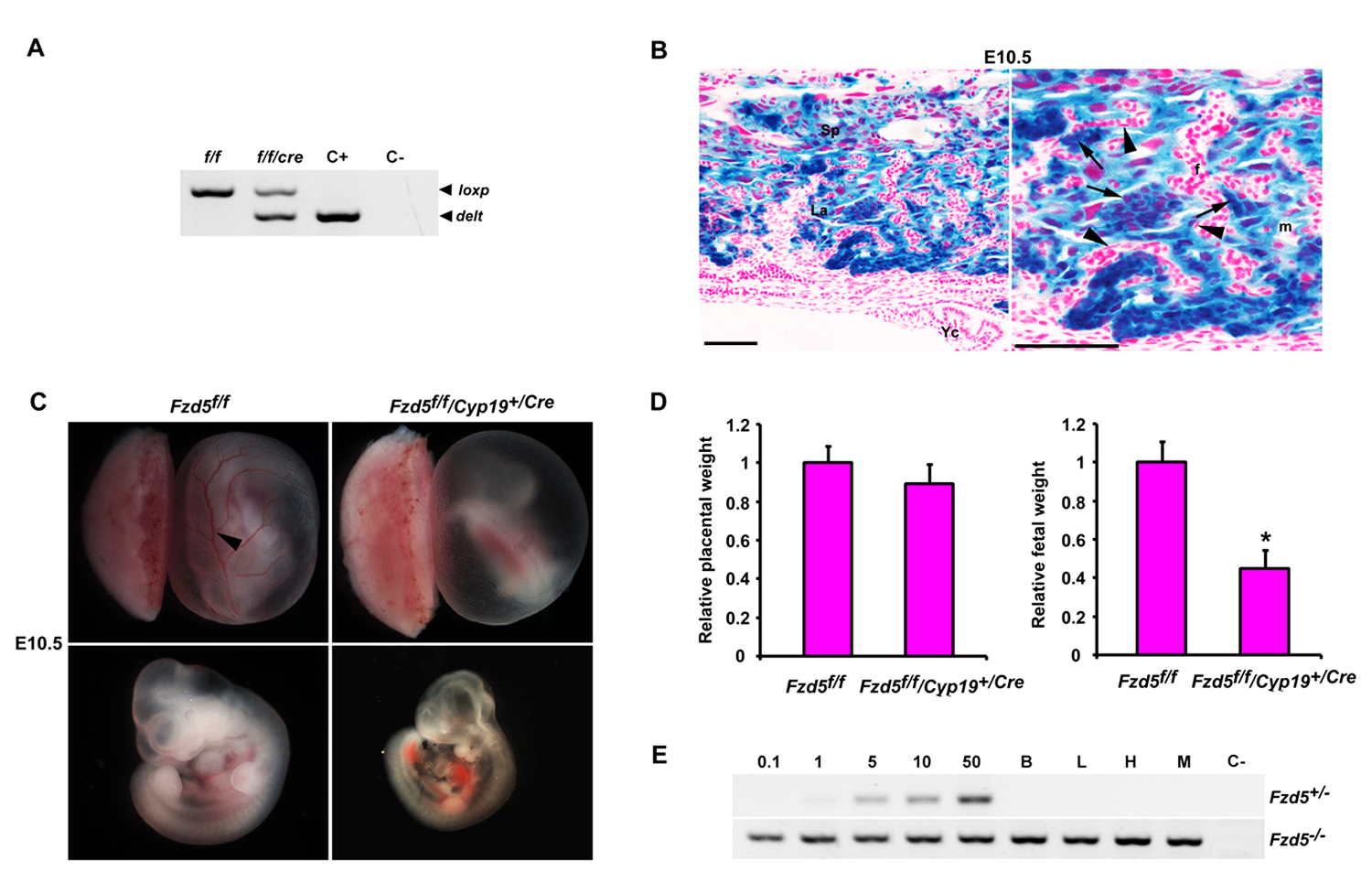

Supplement: Figure S3 — Conditional deletion of Fzd5 gene in trophoblasts during placental development in mice. (A and B) The efficiency of conditional deletion of Fzd5 by Cyp19-Cre in trophoblast cells was detected by RT-PCR (A) and LacZ staining of placentas from Rosa26loxp/loxp mice and Cyp19-Cre +/− mice intercross (B). Fzd5 gene could be selectively deleted in the trophoblast cells (arrows), while no depletion of Fzd5 in embryonic tissues (fetal endothelial cells) (arrowheads) and yolk sacs was observed. (C) Whole mount views of E10.5 control (Fzd5f/f) and Fzd5 conditional-deleted (Fzd5f/f/Cyp19+/Cre) placentas and yolk sacs. Trophoblast specific deletion of Fzd5 led to fetal growth retardation and pale yolk sacs with no blood perfusion. (D) Placental and fetal weight after Fzd5 conditional deletion by Cyp19Cre at E10.5. While the placental weight was not affected by Fzd5 conditional deletion, the fetal weight was reduced significantly (P<0.05). (E) PCR of genomic DNA from tetraploid rescued embryos as well as mixed DNA templates showing that wild-type tetraploid cells were excluded from the embryo proper. Numbers above the lanes represent the percentage of wild-type DNA in the wild-type/Fzd5 mutant mixed DNA templates. f, fetal vessel; La, labyrinth; m, maternal blood sinus; Sp, spongiotrophoblast layer; Yc, yolk sac; B, brain; L, lung; H, heart; M, muscle. Scale bars: 200 µm. (TIF) [file pbio.1001536.s003.tif]

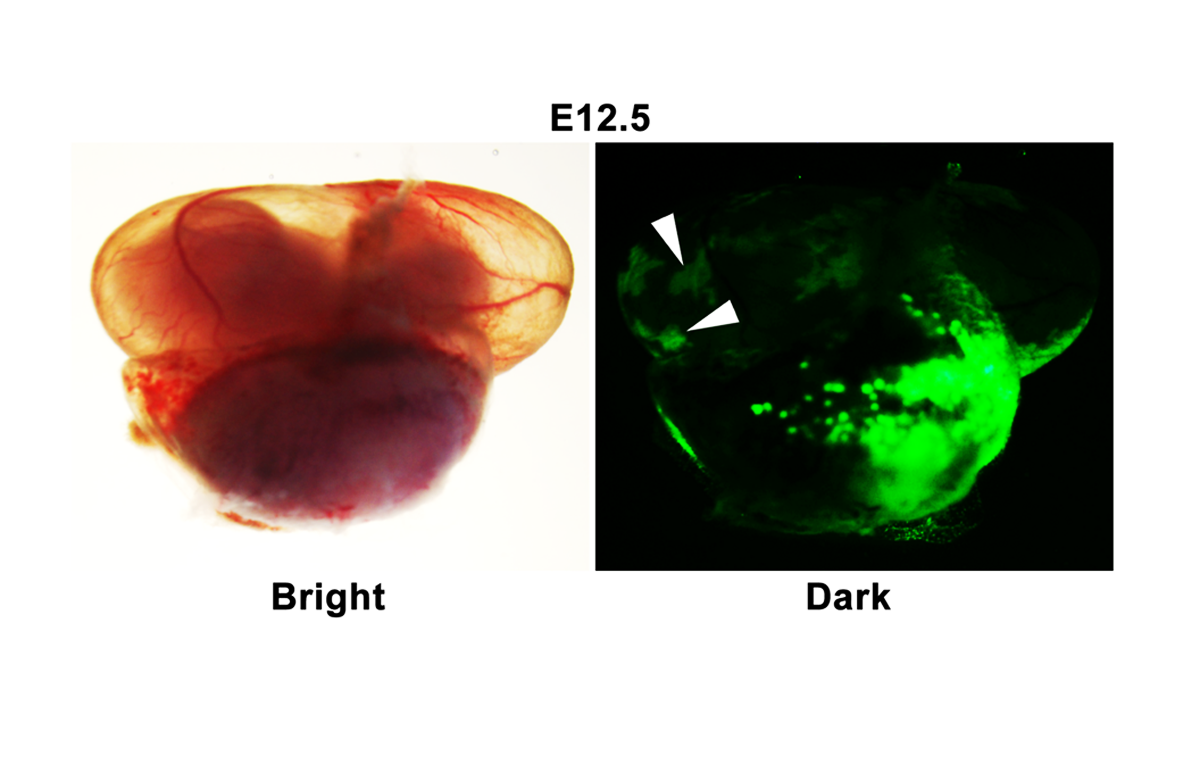

Supplement: Figure S4 — Tetraploid trophoblast with EGFP contributed exclusively to the endoderm of the yolk sacs. E12.5 fetus within its yolk sac was with intact placenta generated by tetraploid aggregation after complementation with wild-type tetraploid Egfp +/− embryos. Note that the contribution of tetraploid trophoblast cells with EGFP to the endoderm of the yolk sac (arrowhead). (TIF) [file pbio.1001536.s004.tif]

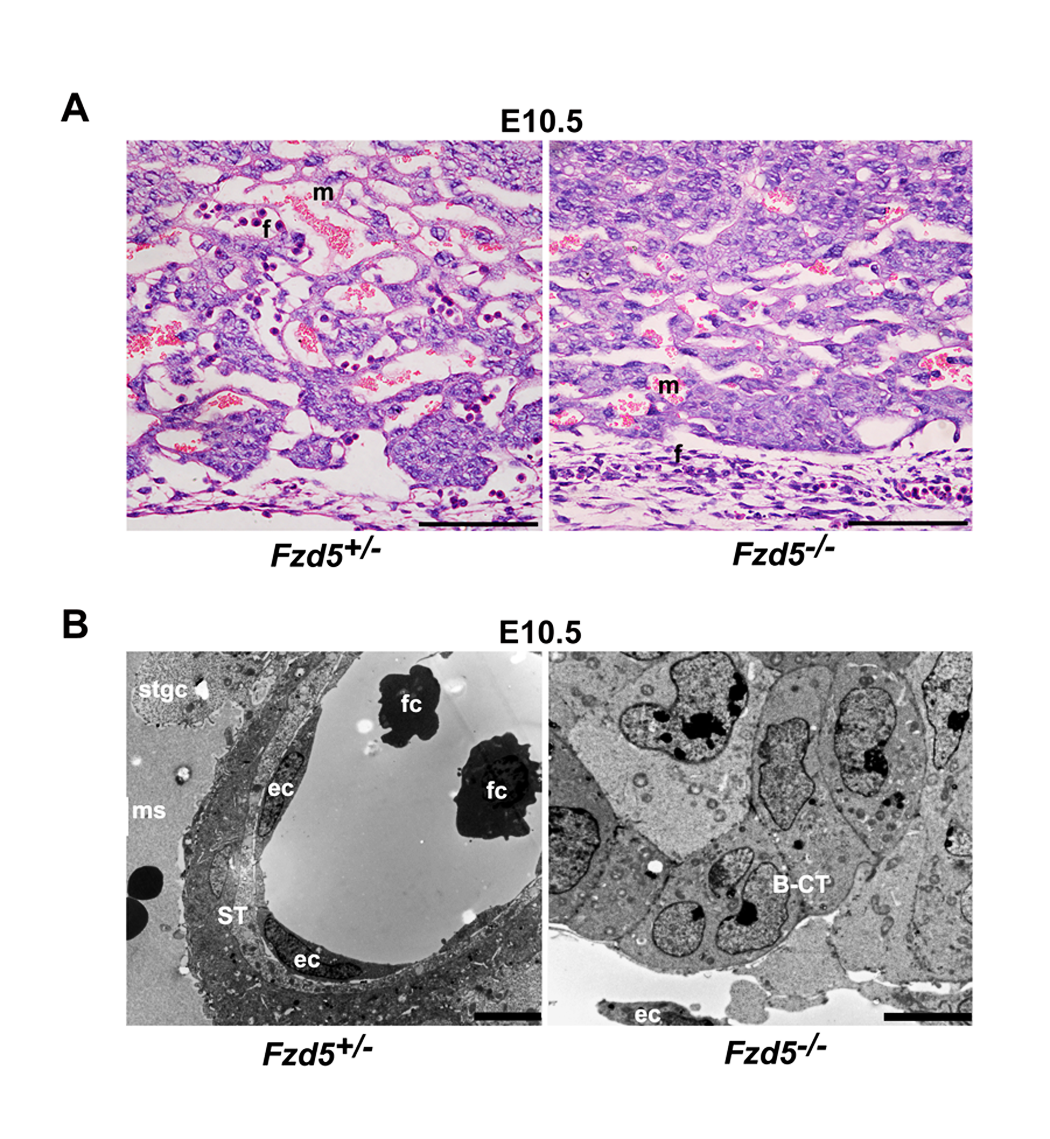

Supplement: Figure S5 — Impaired labyrinth formation caused by Fzd5 deletion. (A) HE staining of E10.5 control (+/−) and Fzd5-null (−/−) placentas. Note that the fetal vessel didn't penetrate into the chorion to interdigitate with the maternal sinuses and a functional labyrinth layer failed to form in the Fzd5-null (−/−) placentas. Scale bars: 400 µm. (B) Analysis of chorionic trophoblasts in E10.5 placentas by electron microscopy. While elongation and fusion of chorionic trophoblast cells to form syncytiotrophoblast layer II between the fetal blood vessels and maternal sinus in Control (+/−) placentas, basal chorionic trophoblast cells remain unfused and undifferentiated in Fzd5-null (−/−) placentas. f, fetal vessel; m, maternal blood sinus; B-CT, basal chorionic trophoblasts; ec, fetal endothelial cells; fc, fetal blood cells; ms, maternal blood sinus; ST, syncytiotrophoblast cells; stgc, sinusoidal trophoblast giant cells. Scale bars: 5 µm. (TIF) [file pbio.1001536.s005.tif]

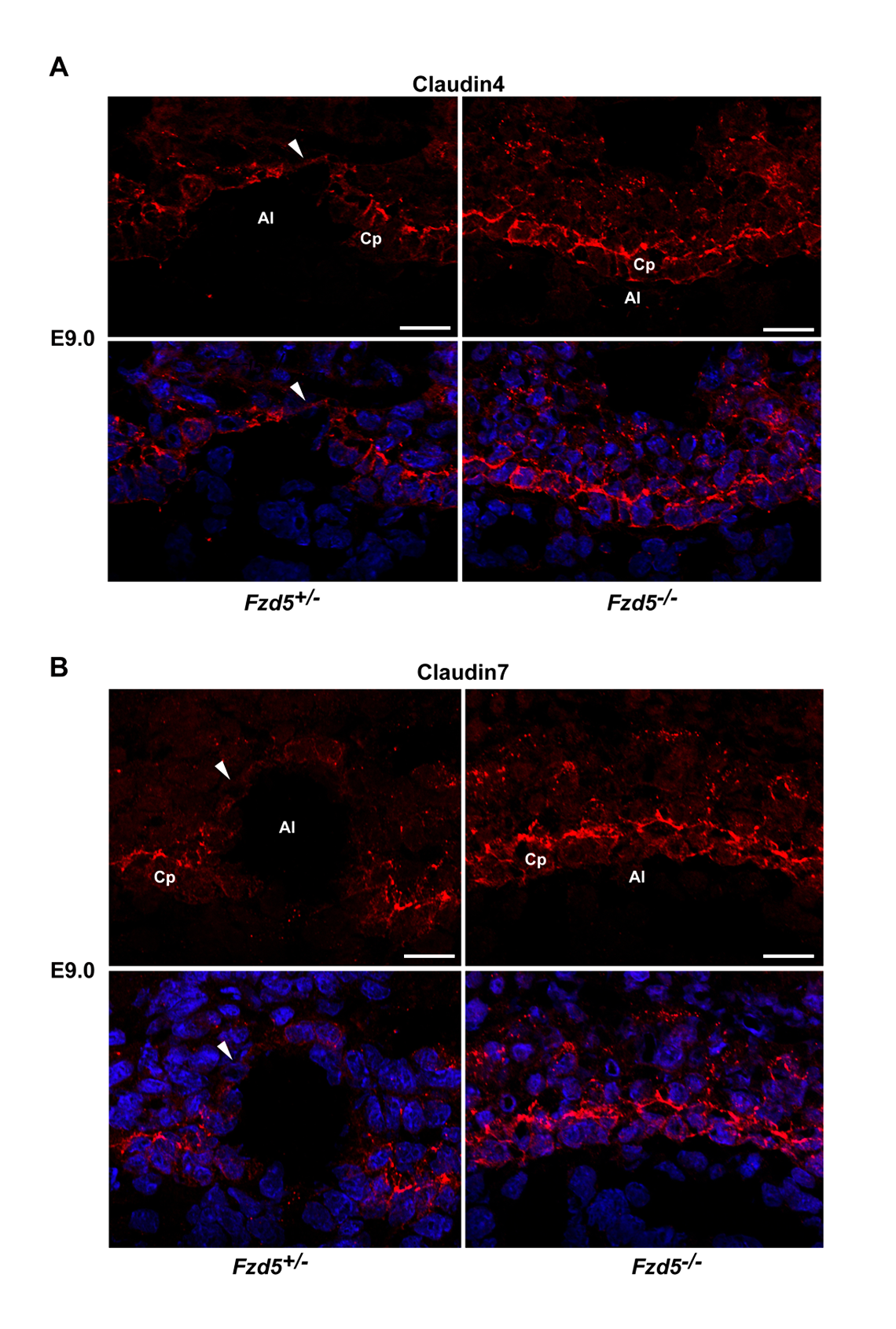

Supplement: Figure S6 — Tight junctions revealed by claudins exist between the trophoblast cells at the basal chorionic plate. (A and B) The expression of Claudin4 (A) and Claudin7 (B) at E9.0 chorionic plate was revealed by immunostaining. Note that the Claudin4 and 7 are mainly expressed at the apical side of the trophoblast cells at the base of the chorionic plate, and their expression was reduced or diminished at the branching sites (arrowheads). Al, allantois; Cp, Chorionic plate. Scale bars: 200 µm. (TIF) [file pbio.1001536.s006.tif]

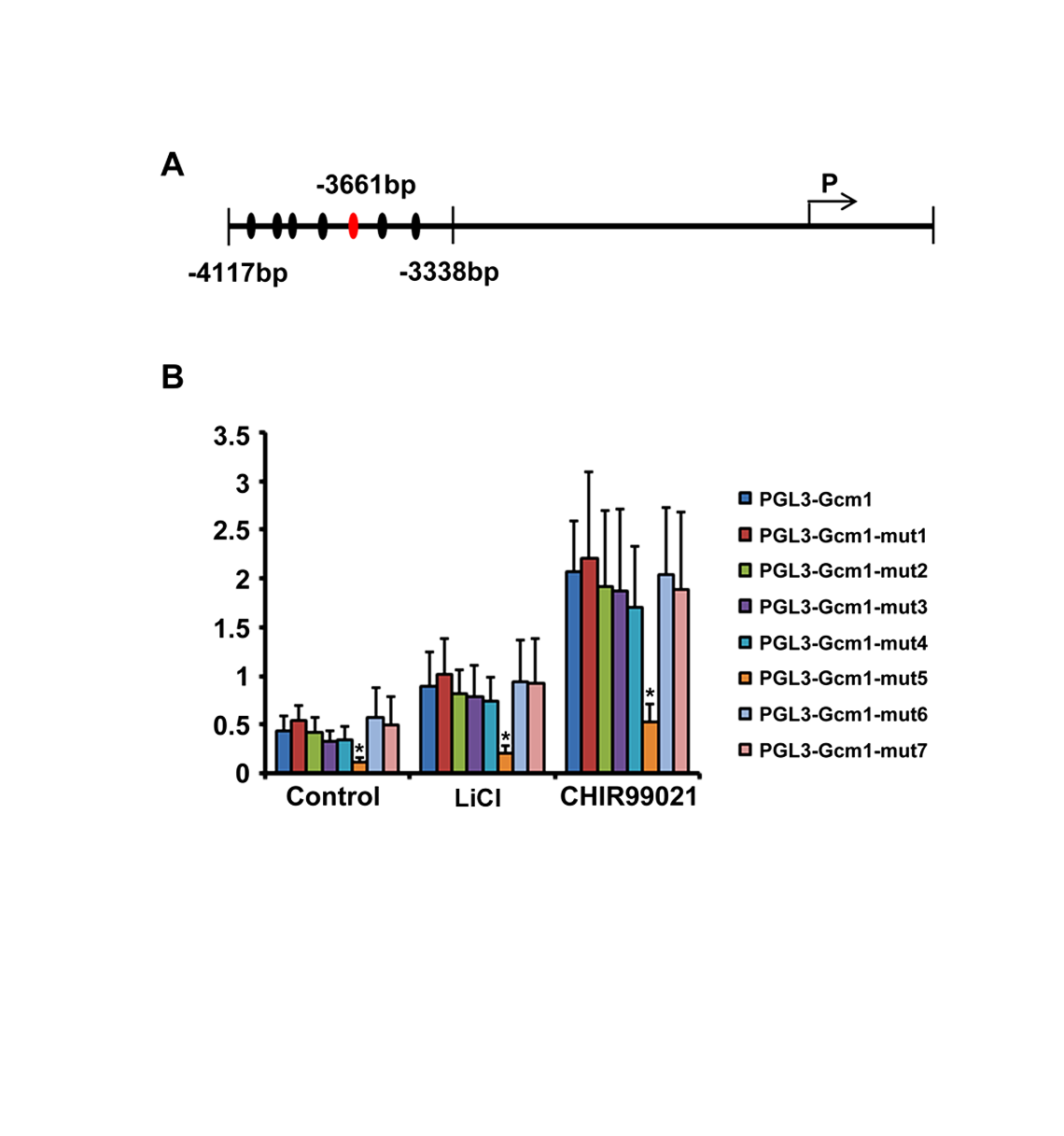

Supplement: Figure S7 — Analysis of LEF/TCF binding sites in the Gcm1 promoter. (A) Map of the Gcm1 promoter, indicating the 5-kb region containing the LEF/TCF binding sites. A fragment containing nucleotides (nt) −4,117 to −3,338 relative to the transcription site (P) is comprised of seven binding sites for LEF/TCF complex. (B) Point mutations of the seven binding sequences revealed that only the fifth sequence was responsive to canonical Wnt pathway agonists, LiCl and CHIR99021 (red in A). (TIF) [file pbio.1001536.s007.tif]

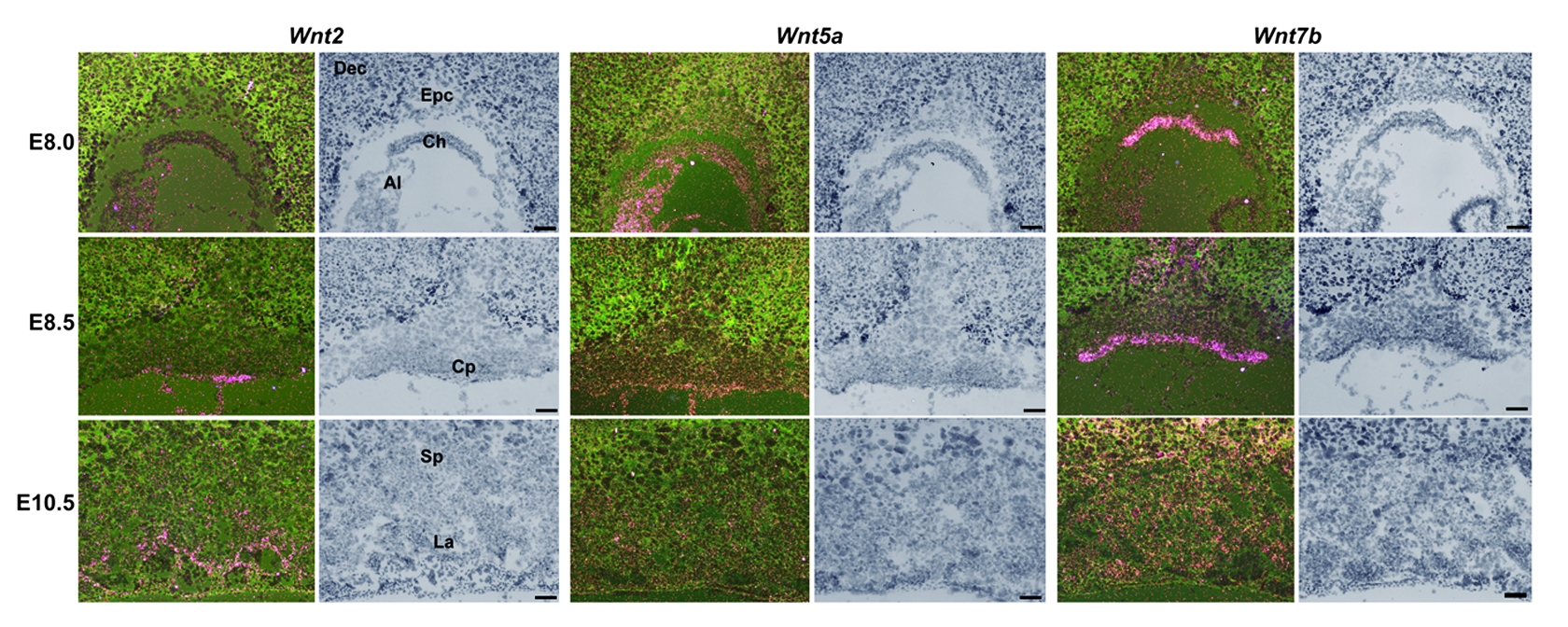

Supplement: Figure S8 — The expression of Wnt2, Wnt5a, and Wnt7b was detected by in situ hybridization during early placentation. Wnt2 was expressed in the allantois before chorioallantoic attachment at E8.0 and was localized to the endothelial cells of the fetal blood vessels at later stages. While Wnt7b was localized to the base of the chorion plate, Wnt5a was detected in both the chorion and allantois. Al, allantois; Ch, Chorion; Dec, decidua; Epc, ectoplacental core; La, labyrinth layer; Sp, spongiotrophoblast layer. Scale bars: 200 µm. (TIF) [file pbio.1001536.s008.tif]
