# Supplementary material for: A Positive Feedback Loop Involving Gcm1 and Fzd5 Directs Chorionic Branching Morphogenesis in the Placenta
Source: PLoS Biol. 2013 Apr 16;11(4):e1001536. doi: 10.1371/journal.pbio.1001536 (PMC3627642; doi:10.1371/journal.pbio.1001536)
Supplement: Table S1 — Oligonucleotides. (DOC) [file pbio.1001536.s009.doc]

**Table S1** Oligonucleotides

| **Quantitative RT-PCR primers,** Sequence (5’→3’) | |
| --- | --- |
| Ascl2-F (CAGCTGCGAGGGAGAGCTAA) | Ascl2-R (GATGCTCAGTAGCCCCCTAACC) |
| Cdx2-F (ACCGGAATTGTTTGCTGCTGT) | Cdx2-R (TCCCGACTTCCCTTCACCAT) |
| Cebpa-F (AAAGCCAAGAAGTCGGTGGAC) | Cebpa-R (CTTTATCTCGGCTCTTGCGC) |
| Dlx3-F (TCGCCCAAGTCGGAATATACC) | Dlx3-R (AGTAGATCGTTCGCGGCTTTC) |
| Eomes-F (GGTGTACAACAGCGCTTGCA) | Eomes-R (CCATGCCTTTGGAGGTGTCTT) |
| Esrrb-F (GCAGTCCTTCGTGCTGTCTCA) | Esrrb-R (TACATGGGACTGGATGGGAGAT) |
| Esx1-F (AGGAACCCGAGGGATTCGA) | Esx1-R (GAAGCCGCCTCCAAGGTTT) |
| hFZD5-F (CTTGTTTCCAAAGTCCAATCAAGTG) | hFZD5-R (GCCTACTCTTCACCCTTCTTTAACG) |
| GAPDH-F (TGGCAAAGTGGAGATTGTTGCC) | GAPDH-R (AAGATGGTGATGGGCTTCCCG) |
| mGcm1-F (CCCCAGCAAGTTCCATCAGA) | mGcm1-R (AAGGCTCACCTCCCGGATT) |
| hGCM1-F (GGTGCTTGAGTGGGCCGATCC) | hGCM1-R (AGGTGCCGCTGCGCATTCTT) |
| Hand1-F (GGGTTAAACCCGGTCTTTGG) | Hand1-R (AAGGACCTGCCGACCTCTTG) |
| Itga4-F (AATCTCCTCCACCTACTCACAGAG) | Itga4-R (ACCAACGGCTACATCAACATATCC) |
| Pl-1-F (CAGGCTCCGGAATGCAATT) | Pl-1-R (GCAGTTGGTTTGGAGGACACA) |
| Syna-F (AGATACCCCGATGACCACGTC) | Syna-R (TGAGGATCGTCTGGGTGGAG) |
| Synb-F (CCACCACCCATACGTTCAAA) | Synb-R (GGTTATAGCAGGTGCCGAAG) |
| Syncytin1- F (GGAGTATCCCAGGCGTTAGG) | Syncytin1-R (TTGGGTCAAGCACAAGGTCA) |
| Tfeb-F (TGGACTCCTGCAGTCACTGAAG) | Tfeb-R (AGCTGAGCCATGAACCCAAGT) |
| Tpbpa-F (CAGAGAGTGGCGATGGGTTTT) | Tpbpa-R (GACAATGGCACAGTGGCTGTT) |
| hWNT2-F (TGGCTCCCTCTGCTCTTGAC) | hWNT2-R (CTGGCACATTATCGCACATCAC) |
| Vcam1-F (GTGACTCCATGGCCCTCACTT) | Vcam1-R (CGTCCTCACCTTCGCGTTTA) |
| **Primers for Gcm1 promoter** | |
| Gcm1-P-F (TGGTAGAGTGGATTATAGG) | Gcm1-P-R (AAGACACGTATATCTTCCG) |
| **siRNA oligonucleotides** | |
| Stealth RNAi (Invitrogen) |  |
| siFZD5-1 | GAGCACAACCACAUCCACUACGAGA |
| siFZD5-2 | GCACCAUCGUCUUCCUCCUGGUCUA |
